# Decision-Making and the Contract of the Complementary Product Supply Chain Considering Consumers’ Environmental Awareness and Government Green Subsidies

**DOI:** 10.3390/ijerph19053100

**Published:** 2022-03-06

**Authors:** Lingzhi Shao, Qianwen Liu

**Affiliations:** School of Management Science and Engineering, Anhui University of Technology, Ma’anshan 243002, China; qianwen2022@126.com

**Keywords:** complementary products, green decisions, contract coordination, consumers’ environmental awareness, green subsidy

## Abstract

The environmental awareness of consumers and enterprises has gradually increased, and green production and green consumption have become the main theme of social economy. On the other hand, the complementary product market has become an important source of competitive advantage for enterprises. Considering a complementary product supply chain, and taking account of the consumers’ environmental awareness and the green subsidies provided by the government, this paper examines members’ decisions in relation to four contract models based on game theory. By solving the model, it is shown that the government’s green subsidy plan improves the green degree of subsidized products and complementary products. Furthermore, compared to wholesale price contracts, revenue-sharing and cost-sharing contracts motivate manufacturers to improve the greenness of subsidized products, and they achieve a Pareto improvement for the whole supply chain and its members, when the contract parameters are appropriate. Numerical experiments also reveal that both the greenness of the complementary products and the profit for members increase with the green innovation spillover effect as a result of the complementary products and the scale of green consumers with environmental awareness in the market. This study provides good guidance for decision-making concerning the complementary product supply chain, and further contributes to environmental protection.

## 1. Introduction

With the proposal of the concept of low carbon and emission reduction, consumers’ awareness of environmental protection and low carbon is also improving. Low-carbon products with a high price are gradually favored by consumers, and even become the first choice of some consumers. In 2020, the sales of new energy vehicles in China increased by 10.9% over the previous year, and household appliances, such as washing machines, refrigerators and water heaters marked with energy efficiency labels, also became popular with consumers. According to e-commerce enterprise (Pinduoduo) statistics, in 2021, the consumption population and amount of energy-saving household electricity on the platform increased by 35% and 41% year on year; among similar household electrical products, the consumption amounts of energy-saving products accounts for about 63%.

As people pay more and more attention to environmental problems and environmental protection, green manufacturing has become an inevitable choice for economic and social development. Enterprises concentrate on the green innovation of products and green supply chain management as essential operational practices. SAIC GM launched the “green future” strategy in 2008, which aims to standardize and advocate its multiple upstream and downstream suppliers to go hand in hand to create high-tech green products with “better performance, lower energy consumption and less emission”, and released a number of hybrid environmental protection models in 2017. Epson advocates green printing and continuously innovates in the field of inkjet and vision. It is committed to reducing carbon dioxide emissions in the life cycle of all products and services by 90% by 2050. Additionally, Huawei, Lenovo and Apple are constantly working on constructing a green supply chain, and have achieved good results. It is found that green supply chain management and strategic green marketing orientation have positive and significant effects on green consumption intention, and environmental concern (i.e., green image) partially mediates the relationship between strategic green marketing orientation, green supply chain management and green consumption behavior [1].

At the same time, green supply chain management has received extraordinary attention from government departments. In 2017, “The Guidance on Actively Promoting Supply Chain Innovation and Application”, issued by the General Office of the State Council of the People’s Republic of China, explicitly advocated and built green supply chains by vigorously promoting green manufacturing and circulation. In the distribution chain, governmental departments in many countries provided various subsidy policies to encourage green production and stimulate the sale and consumption of green products. For example, many countries issued financial support policies to promote and apply new energy vehicles by subsidizing the consumers who purchase new energy vehicles. To support the sales of green and intelligent home appliances, the governments provide appropriate subsidies for consumers to buy new green and smart home appliances with long industrial chains, significant driving coefficients, and noticeable synergistic effects of energy saving and emission reduction. In addition, the enterprises that purchase special equipment from environmental protection catalogs could enjoy tax preference in many countries.

In practice, with the refinement and globalization of consumption and production, the role of complementary product strategy has become increasingly prominent, and the complementary product supply chain has become an important operation for enterprises. With iTunes, the world’s largest music store (an extremely effective complementary product supporting the basic product, iPod, which launched in 2001), Apple, finally occupied 70% of the MP3 market in the United States. Leica, a famous German camera brand, chose to join hands with Huawei in 2016, trying to cope with the overall decline in the camera industry by changing the previous product strategy and using complementary advantages. By starting with an acknowledgement of the needs of users, IKEA skillfully connected furniture and catering, two areas that do not seem to be directly related to each other. This product strategy of driving the original low-frequency furniture with high-frequency catering is also a complementary product strategy. However, complementary products (such as automobiles and on-board equipment, automobile services, printer and ink cartridges, paper and other consumables, home appliances and related products, cameras and films; and lamps and bulbs) have mutual influence and spillover effects in investment decision-making concerning emission reduction technology, low-carbon product design and green sales operations. Enterprises in the supply chain of complementary products may share the market performance caused by green and low-carbon products. For example, when computing hardware (Intel processor) suppliers invest in emission-reduction technology to reduce carbon emissions in the production process, improve the degree of environmental protection of products, and then improve the market demand of computers, computer software (Microsoft operating system) suppliers and computer vendors profit from the increase in market demand without any investment. Then, free riding occurs. Therefore, in the face of consumers with environmental awareness and government green subsidies, the research on games between complementary product enterprises and the coordination of supply chains is very important.

In this paper, we seek to analyze the price and green innovation game decisions in the supply chain of complementary products under the scenario of government green subsidies to retailers. Furthermore, we also investigate the contractual parameter settings among supply chain members and the coordinated optimization of the supply chain. To address these questions, we consider a complementary product supply chain, including a complementary product supplier, a common retailer, and consumers with environmental awareness, and present four classes of two-period dynamic models based on different contracts, including a wholesale price, subsidy sharing, cost sharing and revenue sharing, taking account of government green subsidies. By solving these problems, we obtain the equilibrium price and green decisions under different contracts and the conditions with which the contract improves the profit of members and the supply chain. We find that it is best for the supply chain to use a revenue sharing contract, when the sharing ratio is appropriate, and then the cost sharing contract is better for the Pareto improvement than the wholesale price and subsidy sharing. In addition, the green spillover effect of complementary products affects the decisions of the manufacturer and retailer.

The rest of the paper is organized as follows: Section 2 reviews the related literature and explains our contributions in more detail; Section 3 outlines the key elements of our model, as well as the derivation of consumer purchase behavior; Section 4 describes the model framework, presents the optimal equilibrium solutions in different cooperation contracts, and reports our main findings; Section 5 investigates the comparison of the different contracts and the effect of factors using numerical experiments; and Section 6 summarizes our conclusions and suggests opportunities for future research.

## 2. Literature Review

The problem of complementary products arises in the field of marketing, which is mainly manifested as the bundle sales of complementary products and the pricing problem as a result of this sales strategy. Venkatesh and Kamakura (2003) [2] studied the optimal pricing of complementary products in the monopoly market under different sales strategies, and analyzed the effects of the marginal cost level and degree of complementarity on three pricing strategies: pure separate pricing, pure bundle pricing and mixed bundle pricing. Chris et al. (2013) [3] studied the bundling pricing and advertising investment of complementary products based on the linear price elastic demand function. The study found that when the price discount is enough to attract customers and the complementarity between the products is adequate, advertising, while implementing the bundling strategy, can improve the enterprise’s operation performance. Gwon et al. (2015) [4] established a competition model between a multi-complementary product enterprise and a single product enterprise to study the hybrid bundling strategy among enterprises. Halmenschlager and Mantovani (2017) [5] studied the impact of bundling on enterprises and social welfare. The conclusion shows that although the mixed bundling strategy of complementary products may produce the prisoner’s dilemma, it is still the leading strategy for multi-product enterprises, and it may maximize social welfare when the cost savings generated by creating bundles are large enough.

A number of literatures studied the bundling and pricing decision of the complementary product supply chain. Wei et al. (2012) [6] studied the pricing problem for the structure of a two-level supply chain composed of two complementary product manufacturers and a common retailer. Bhargava (2012) [7] found that the price coordination among supply chain members can make up for the economic benefits lost by bundling sales due to channel conflict to a certain extent. Chakravarty et al. (2013) [8] compared and analyzed bundling and pricing decisions in three supply chains with different coordination degrees. Wei et al. (2015) [9] discussed the optimal price and shelf-life strategy of two complementary products between two manufacturers and one retailer from the perspective of the two-stage game theory. Giri et al. (2016) [10] studied the pricing strategy in a supply chain, including three suppliers providing complementary and competitive products and a common retailer. Wang et al. (2017) [11] studied the pricing decision of complementary products in a dual channel supply chain. Dehghanbaghi and Sajadieh (2017) [12] studied the joint optimization of production, inventory, transportation and pricing in the centralized and decentralized supply chain of complementary products by establishing a mixed integer linear model and compiling an accurate algorithm. It was found that when the correlation between the complementary products changed, the profit of the centralized supply chain was more stable than that of the decentralized supply chain. Shao and Li (2019) [13] studied the impact of the bundling strategy on the complementary product supply chain in relation to competitor strategy and enterprise profit according to different sales strategies and product quality strategies. Giri et al. (2020) [14] studied the pricing decision of complementary products under different supply chain structures. Unlike the above literatures, this paper studies the decision-making concerning the complementary product supply chain in relation to the case of green supply chain management, including the green manufacturing of products and government green subsidies. In addition to pricing decisions, our results show the product’s greenness, the decision of complementary product manufacturers in relation to different supply chain contracts, and the optimal green subsidies sharing ratio of retailers. Additionally, our study found that the decisions are affected not only by the cross-price elasticity of complementary products, but also the spillover benefits of green production and the green subsidy rate of the government. For example, according to the corollaries in this paper, the greenness of the subsided products increases with the green subsidy rate, while the prices of the subsided products decrease with the subsidy rate and the prices of complementary products increase with that the subsidy rate.

To date, there is also limited literature on the supply chain decision-making process of complementary products in the context of the green supply chain, and they still pay attention to the pricing strategy of complementary products. For example, Shan et al. (2020) [15] studied the pricing strategy in the supply chain of complementary products by using the Steinberg game. The study found that the higher the green preference of consumers for one product, the more it can promote another supplier of complementary products to improve the level of green manufacturing. Dobson et al. (2020) [16] demonstrated the possibility of producers controlling the supply of essential complementary components that enter the assembly of competitively produced composite finished goods. They also demonstrated the ruinous effect of independent strategic delegation to managers of influential complementary product producers. In this study, we also consider that a complementary product supply chain consists of manufacturers who supply complementary products to a common retailer. However, different from the above research, we take account of the green spillover effect of complementary products in the product demand model. Because of the green spillover effect, in our results, the green subsidies for one product have a positive effect on the other complementary product’s green decision and performance. Moreover, the study shows that the positive effect of green subsidies increases with the green spillover effect of complementary products. More importantly, we conduct contract models and study the cooperation of supply chains under government green subsidies, including the wholesale price, subsidy sharing, cost sharing and revenue sharing. Our propositions and corollaries present the conditions for the Pareto improvement of a supply chain contract. Moreover, through numerical simulation and comparing finances, we found that the coordination effect of the revenue-sharing contract is the best, and the cost-sharing contract is better than the wholesale-price contract.

Over the last few decades, there has been a worldwide realization of the importance to protect the environment. Many available studies have drawn attention to “Green products” that seek to protect or enhance the environment during production, use, or disposal, by conserving resources and minimizing the use of toxic agents, pollution, and waste [17]. They offer high quality and low overall costs to the consumer and society. Over the last twenty years, systematic reviews for green product innovation, green product development, and green products have been undertaken in the literature. Bhardwaj et al. (2020) [18] used bibliometric tools and various indicators to discern research progress in the field of green products over the period of 1964–2019. The literature closely related to this paper are about the decisions of the green supply chain in which the green products are manufactured and supplied. Firstly, part of the research analyzed and found that the environmental preference of consumers has a greater impact on the green production and emission reduction strategies of supply chain members. Kim and Sim (2016) [19] used the differential game analysis to find that consumers’ low-carbon awareness plays an important role in environmental protection. Enterprises can increase profits by attracting consumers to be more willing to produce low-carbon products. Basiri and Heydari (2017) [20] researched and showed that with the enhancement of consumers’ low-carbon awareness, they are more willing to pay higher prices for low-carbon products, which encourages enterprises to produce green products. Based on the dual channel supply chain, Ji et al. (2017) [21] found that it is more profitable for manufacturers and retailers to jointly take charge of carbon emission reduction based on consumers’ low-carbon preferences. Fan et al. (2017) [22] established the Stackelberg game model and Vertical Nash model from the perspective of static game and dynamic game, respectively, and discussed the effects of retailer altruism preference, consumer low-carbon preference and decision-making parameters on the complex nonlinear dynamic behavior of the two models. Some scholars also consider consumers’ low-carbon preference and emission reduction policies. Du et al. (2016) [23] found that under the restriction of carbon trading, consumers’ low-carbon awareness has an important impact on enterprise production. Hammami et al. (2018) [24] considered how consumers’ low-carbon preferences and the government’s carbon policy affect the enterprises’ pricing, production and emission reduction strategies.

Secondly, green subsidies from the government have also attracted the attention of many researchers. For example, Shi and Min (2015) [25] and Cao et al. (2018) [26] studied the impact of government per-product subsidies versus one-time subsidies given to manufacturers on supply chain decisions. The former concluded that the unit subsidy model is more effective. However, the latter study found that one-time subsidies are more effective in the single-sales channel model, while price subsidies are more influential in the dual-channel model. Yu et al. (2016) [27] studied the decision making of manufacturers producing green products under the consideration of government subsidies. In addition to the single-product supply chain, Sheu et al. (2012) [28] focused on the competitive market and conducted a three-stage game model to study the impact of taxes and subsidies on the green supply chain competition and the competitive decisions among green supply chain subjects. Ashkan (2017) [29] studied the price energy-efficiency competition and cooperation models of two green supply chains (GSCs) under a government financial intervention. Hong et al. (2021) [30] investigated two subsidy policies (subsidy for firm and subsidy for consumer policies) and their impacts on a market that comprises two vertically differentiated products: green and less-green products. Moreover, in relation to the closed-loop supply chain, Liu et al. (2021) [31] established a two-echelon supply chain consisting of a brand owner and an original equipment manufacturer (OEM) to examine the members’ operation strategies and investigate how the government optimizes the levels of the unit subsidy and disposal fee by minimizing the deposit-refund policy deficit.

Finally, many researchers studied the coordination of green supply chains in the case of government subsidies. For example, Yi and Li (2018) [32] explored the coordination effect of cost-sharing contracts on supply chains in the case of government subsidies and carbon taxes on manufacturers producing energy-efficient and emission-reducing products, and found that supply chain contracts can increase manufacturers’ energy savings and improve the profits of the overall supply chain. Li et al. (2021) [33] formulated and analyzed three Stackelberg game models to compare the impacts of two types of subsidies (based on green technology investment cost or the amount of emission reduction) on green technology investments and green marketing coordination. Another two-part tariff (TPT) contract involving government intervention, in terms of taxes or subsidies, was proposed by Zhang et al. (2020) [34], who demonstrated that the proposed TPT contract can achieve global supply chain optimization and aid in achieving green improvement. They further illustrated that the optimal green improvement degree is influenced by green technology investments, government interventions, and the additional demand from customer green preferences. Liu et al. (2021) [35] investigated the cooperative relationships in a three-party sustainable supply chain (TSSC) and the coordination of a supply chain utilizing the Nash equilibrium strategy. Liu et al. (2021) [36] constructed a master–slave game theory model for a supplier and a manufacturer and analyzed the effect of suppliers’ eco-designs on the economic benefits of the up-downstream supply chain and the mechanisms. In the above literature, the government provides green subsidies to manufactures. However, consumers’ environmental awareness and purchase desire have an important impact on the operation and income of the supply chain. Therefore, the government often provides subsidizes to consumers with green purchase behavior. Wang et al. (2021) [37] investigated a green supply chain (GSC) in the context of government subsidies for consumers, and examined the value of information sharing on the decisions of the GSC in three cases: centralized decision, and decentralized decision with and without demand forecast information sharing between the retailer and the manufacturer.

In summary, scholars paid more attention to single or competitive product supply chains. However, unlike ordinary products, as shown in the conclusion of our article, the relationship between complementary products not only affects the pricing decision of the supply chain, but also affects the green manufacturing of products and the profits of the green supply chain. In addition, unlike the above literature focusing on the green subsidies presented to manufacturers, we consider the subsidies presented to sellers, which are important and common in green product operations. On the one hand, the sellers are encouraged to invest more in green marketing to increase the market demand for green products and improve the performance of the supply chain. On the other hand, sellers are willing to share the profits or green manufacturing costs with manufacturers to a certain degree. Therefore, as our results show, the Pareto improvement is attained in the retailers’ sharing contracts.

## 3. Model

**Figure 1:**
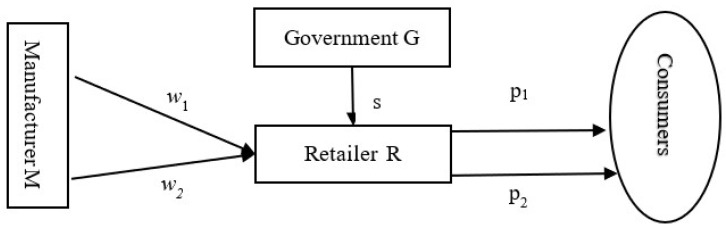
Structure of the complementary products supply chain.

This paper considers a two-stage supply chain, including a complementary products manufacturer (M) and a common retailer (R). We develop Stackelberg game models to analyze the optimal decisions of supply chain members and the contractual coordination of the supply chain considering government green subsidies. The models utilize the manufacturer of the complementary products as the dominant player in the game and the retailer as the follower. As an external supply chain player, the government presents an appropriate green subsidy rate to the retailer’s green products. Figure 1 shows the supply chain structure.

The manufacturer produces two complementary products: green products 1 (such as a computer mainframe) and green products 2 (such as computer monitors and network devices), and sells them to the common retailer simultaneously. To reduce notation and without a further loss of generality, we assume that products 1 and 2 have complete complementary symmetry and are completely symmetrical in the market demand, as well as being cost coefficient. Relative to asymmetric complementary products, such as washing machines and laundry fluids, cars and seat covers, symmetrical complementary products, such as printers and ink cartridges, computers and network equipment, contribute almost equally to consumers’ consumption values. The green degree (greenness) of products 1 and 2 are assumed to be $e_{1}$ and $e_{2}$. The green degree of products can be used to evaluate the economic rationality, resource effectiveness and environmental coordination of green products. Moreover, the product green degree can refer to the degree of friendliness of products to the environment or the comprehensive evaluation of the green, economic and advanced technologies of the products. Therefore, in comparison to ordinary products, the higher the technical and environmental coordination of the green products, the higher the greenness. In this paper, we assume that the greenness of a product is determined by the manufacturer’s green technology investment. Therefore, the green products are costly because of the green technologies or the introduction of new equipment reducing the environmental impact of the manufacturing process. By referring to [27,33], it can be observed that the green production costs faced by the manufacturer are $\frac{\eta }{2}e_{1}^{2}$ and $\frac{\eta }{2}e_{2}^{2}$, respectively, where η is the manufacturer’s green manufacturing coefficient. Similar to Dobson et al. (2020) [16], we set the production cost of the complementary products to zero. The retailer offers complementary products to consumers at retail prices $p_{1}$ and $p_{2}$, and receives green subsidies from the government. This paper considers the government’s green subsidy at a fixed rate for green products 1 only to the retailer, and this subsidy is linearly related to the greenness of products 1, i.e., the higher the greenness, the higher the subsidy, according to [33]. The unit product subsidy received by the retailer is $se_{1}$, where s (s>0) is the government’s green subsidy coefficient per unit product.

In the consumer market, similar to the studies [23,29], the market demand is assumed to be a linear function of the product price and greenness, where the consumers’ environmental awareness is considered. Unlike general products, the market demands of complementary products interact with each other in terms of the cross-price elasticity coefficients of the complementary products [15,16], and there is a specific spillover effect of the complementary products’ greenness. The high greenness of a household appliance or electronic equipment shows that the related complementary products from the same manufacturer are more likely to be green, increasing the purchase intention due to the spillover effect of the complementary products’ greenness. Therefore, the market demands for two symmetrical complementary products are assumed as $D_{1}=a-p_{1}-\alpha p_{2}+e_{1}+\beta e_{2}$; $D_{2}=a-p_{2}-\alpha p_{1}+e_{2}+\beta e_{1}$, where a is the initial market potential, α is the cross-price elasticity coefficient of the complementary products, and β is the green spillover coefficient of the complementary products, assuming 0<α<1, 0<β<1. Assuming that consumers with environmental awareness are more sensitive to the green degree of complementary products than the price of complementary products, then the green manufacturing spillover effect of complementary products is greater than the cross-price elasticity coefficient (β>α).

To analyze the game relationship between members and the coordination of the supply chain, this paper considers four kinds of contracts between manufacturers and retailers: wholesale price, subsidy-sharing, revenue-sharing, and cost-sharing contracts. Then, we construct and solve game models to obtain the optimal pricing and greenness decisions of supply chain members, respectively. Finally, we investigate the impact of different contracts on the greenness of complementary products, the supply chain members’ profits, and the supply chain optimization and coordination.

## 4. Equilibrium and Comparative Analysis

### 4.1. Centralized Decision Model

To analyze and compare the effects of four contracts on the supply chain optimization and coordination, we firstly considered the centralized model (marked by the superscript c) in which the supply chain as a whole makes centralized decisions of retail prices and greenness. According to the assumptions, the profit function of the supply chain is as follows:

(1) $$\Pi_{sc}^{c}=\left(p_{1}^{c}+se_{1}^{c}\right)\left(a-p_{1}^{c}-\alpha p_{2}^{c}+e_{1}^{c}+\beta e_{2}^{c}\right)+p_{2}^{c}\left(a-p_{2}^{c}-\alpha p_{1}^{c}+e_{2}^{c}+\beta e_{1}^{c}\right)-\frac{\eta }{2}e_{1}^{c2}-\frac{\eta }{2}e_{2}^{c2}$$

The first-order derivative of the profit function with respect to the retail price and product greenness ($\frac{\partial \Pi_{sc}^{c}}{\partial p_{1}^{c}},\;\frac{\partial \Pi_{sc}^{c}}{\partial p_{2}^{c}},\;\frac{\partial \Pi_{sc}^{c}}{\partial e_{1}^{c}},\;\frac{\partial \Pi_{sc}^{c}}{\partial e_{2}^{c}}$) can be obtained and made to equal zero to obtain the optimal green decisions and retail prices of products 1 and 2. Then we can obtain the following proposition 1:

**Proposition** **1.** *In the centralized decision model, the optimal decisions of the supply chain are presented below*.

(2) $$e_{1}^{c*}=\frac{\begin{array}{c} \left[2\left(1-\alpha^{2}\right)\eta -\left(1+\beta^{2}\right)+2\alpha \beta \right]\left[\left(1+\beta \right)\left(1-\alpha \right)a+\left(1-\alpha^{2}\right)as\right]+\\ \left[\left(1-\alpha^{2}\right)\beta s+2\beta -\left(1+\beta^{2}\right)\alpha \right]\left(1+\beta \right)\left(1-\alpha \right)a \end{array}}{\begin{array}{c} {}[2\left(1-\alpha^{2}\right)\left(\eta -2s\right)-\left(1-s^{2}\right)-\left(\beta -\alpha s{)}^{2}\right][2\left(1-\alpha^{2}\right)\eta -\left(1+\beta^{2}\right)+2\alpha \beta ]-\\ \left[\left(1-\alpha^{2}\right)\beta s+\beta \left(1-\alpha \beta \right)\right]\left[\left(1-\alpha^{2}\right)\beta s+2\beta -\left(1+\beta^{2}\right)\alpha \right] \end{array}}\;e_{2}^{c*}=\frac{\begin{array}{c} \left[2\left(1-\alpha^{2}\right)\eta -\left(1+\beta^{2}\right)+2\alpha \beta \right]\left(1+\beta \right)\left(1-\alpha \right)a+\\ \left[\left(1-\alpha^{2}\right)\beta s+\beta \left(1-\alpha \beta \right)\right]\left[\left(1+\beta \right)\left(1-\alpha \right)a+\left(1-\alpha^{2}\right)as\right] \end{array}}{\begin{array}{c} {}[2\left(1-\alpha^{2}\right)\left(\eta -2s\right)-\left(1-s^{2}\right)-\left(\beta -\alpha s{)}^{2}\right][2\left(1-\alpha^{2}\right)\eta -\left(1+\beta^{2}\right)+2\alpha \beta ]-\\ \left[\left(1-\alpha^{2}\right)\beta s+\beta \left(1-\alpha \beta \right)\right]\left[\left(1-\alpha^{2}\right)\beta s+2\beta -\left(1+\beta^{2}\right)\alpha \right] \end{array}}\;p_{1}^{c*}=\frac{\left(1-\alpha \right)a+\left[1-\alpha \beta -\left(1-\alpha^{2}\right)s\right]e_{1}^{c*}+\left(\beta -\alpha \right)e_{2}^{c*}}{2\left(1-\alpha^{2}\right)},\;p_{2}^{c*}=\frac{\left(1-\alpha \right)a+\left(\beta -\alpha \right)e_{1}^{c*}+\left(1-\alpha \beta \right)e_{2}^{c*}}{2\left(1-\alpha^{2}\right)}$$

The expression of the optimal green decisions in proposition 1 implies that the optimal greenness of the products relates to the cross-price elasticity of the complementary products, the green spillover effect, and the green manufacturing input coefficients of the products, and is influenced by the unit green subsidy coefficient of the government. The retail prices of the complementary products positively correlate with the greenness of the complementary products. Further analysis leads to corollary 1.

**Corollary** **1.** *In the case of green subsidies presented by the government, the relationships between the subsidy rate and the greenness of the products are* $\frac{\partial e_{1}^{c*}}{\partial s}>0$*and*$\frac{\partial e_{2}^{c*}}{\partial s}>0$*; and the relationships between the subsidy rate and the retail price of the products are* $\frac{\partial p_{1}^{c*}}{\partial s}<0$*and*$\frac{\partial p_{2}^{c*}}{\partial s}>0$.

**Proof.**  Firstly, reorganize the expression of $e_{1}^{c*}$ as follows:

$$e_{1}^{c*}=\frac{\begin{array}{c} \{\left[2\left(1-\alpha^{2}\right)\eta -\left(1+\beta^{2}\right)+2\alpha \beta \right][(1-\alpha^{2})a+\left(1+\beta \right)\left(1-\alpha \right)a\left(1-\alpha^{2}\right)\beta \}s+\\ {}[2\left(1-\alpha^{2}\right)\eta -\left(1+\beta^{2}\right)+2\alpha \beta ][\left(1+\beta \right)\left(1-\alpha \right)a]+[2\beta -\left(1+\beta^{2}\right)\alpha ](1+\beta )(1-\alpha )a \end{array}}{\begin{array}{c} {}[2\left(1-\alpha^{2}\right)\left(\eta -2s\right)-\left(1-s^{2}\right)-\left(\beta -\alpha s{)}^{2}\right][2\left(1-\alpha^{2}\right)\eta -\left(1+\beta^{2}\right)+2\alpha \beta ]-\\ \left[\left(1-\alpha^{2}\right)\beta s+\beta \left(1-\alpha \beta \right)\right]\left[\left(1-\alpha^{2}\right)\beta s+2\beta -\left(1+\beta^{2}\right)\alpha \right] \end{array}}$$

According to the assumptions, we can easily determine that $\frac{\partial \left\{\left[2\left(1-\alpha^{2}\right)\eta -\left(1+\beta^{2}\right)+2\alpha \beta \right]\left(1-\alpha^{2}\right)a+\left(1+\beta \right)\left(1-\alpha \right)a\left(1-\alpha^{2}\right)\beta \right\}s}{\partial s}>0$, $\frac{\partial [2\left(1-\alpha^{2}\right)\left(\eta -2s\right)-\left(1-s^{2}\right)-\left(\beta -\alpha s{)}^{2}\right]}{\partial s}<0$, and $\frac{\partial \left(1-\alpha^{2}\right)\beta s}{\partial s}>0$. Therefore, $\frac{\partial e_{1}^{c*}}{\partial s}>0$ can be analyzed. Similarly, $\frac{\partial e_{2}^{c*}}{\partial s}>0$ can be obtained. Then, the first part of corollary 1 can be proven.

Secondly, there is $\frac{\partial e_{1}^{c*}}{\partial s}>\frac{\partial e_{2}^{c*}}{\partial s}$, because of $\left\{\left[2\left(1-\alpha^{2}\right)\eta -\left(1+\beta^{2}\right)+2\alpha \beta \right]\left[\left(1+\beta \right)\left(1-\alpha \right)a+\left(1-\alpha^{2}\right)as\right]+\left[\left(1-\alpha^{2}\right)\beta s+2\beta -\left(1+\beta^{2}\right)\alpha \right]\left(1+\beta \right)\left(1-\alpha \right)a\right\}>\{\left[2\left(1-\alpha^{2}\right)\eta -\left(1+\beta^{2}\right)+2\alpha \beta \right]\left(1+\beta \right)\left(1-\alpha \right)a+\left[\left(1-\alpha^{2}\right)\beta s+\beta \left(1-\alpha \beta \right)\right]\left[\left(1+\beta \right)\left(1-\alpha \right)a+\left(1-\alpha^{2}\right)as\right]$. In addition, because $\left[1-\alpha \beta -\left(1-\alpha^{2}\right)s\right]>\left(\beta -\alpha \right)$, is the following applies: $\frac{\partial [1-\alpha \beta -\left(1-\alpha^{2}\right)se_{1}^{c*}}{\partial s}>\frac{\partial \left(\beta -\alpha \right)e_{2}^{c*}}{\partial s}$. Furthermore, because $\frac{\partial [1-\alpha \beta -\left(1-\alpha^{2}\right)se_{1}^{c*}}{\partial s}<0$, we can obtain the following: $\frac{\partial p_{1}^{c*}}{\partial s}<0$. Finally, we can easily determine that $\frac{\partial p_{2}^{c*}}{\partial e_{1}^{c*}}>0$ and $\frac{\partial p_{2}^{c*}}{\partial e_{2}^{c*}}>0$. Therefore, the second part of corollary 1 can be proven. □

According to corollary 1, the greenness of both products increases with the subsidies, and the government’s green subsidy to the supply chain improves their green production. The higher the green subsidy rate per product, the higher the greenness of the product. For the supply chain of the complementary products, the government’s green subsidy for product 1 also improves the greenness of complementary product 2, due to the green spillover effect. Therefore, in practice, the government’s green subsidies for the main products encourages supply chains or enterprises to produce more environmentally friendly main products and complementary products. However, an increase in the subsidy rate decreases the retail price of the subsidized product, but raises the retail price of its complementary product.

### 4.2. Wholesale Price Contract and Subsidy-Sharing Contract

Under the wholesale price contract, the manufacturer as the dominant player in the supply chain determines, firstly, the wholesale prices, $w_{1}$ and $w_{2}$, for products 1 and 2, and the greenness, $e_{1}$ and $e_{2}$, respectively. Then, the retailer follows the manufacturer’s decision to set the optimal product retailer prices, $p_{1}$ and $p_{2}$. Therefore, the profits of the manufacturer and retailer are, respectively, presented below.

$\Pi_{m}=w_{1}D_{1}+w_{2}D_{2}-\frac{1}{2}\eta e_{1}^{2}-\frac{1}{2}\eta e_{2}^{2}$ and $\Pi_{r}=\left(p_{1}-w_{1}+se_{1}\right)D_{1}+\left(p_{2}-w_{2}\right)D_{2}$.

Firstly, solve the first-order derivative of the retailer’s profit function for $p_{1}$ and $p_{2}$, according to the manufacturer’s optimal decision, and set it to zero. Then, the retailer’s optimal responses to the manufacturer’s decisions are as follows:

(3) $$p_{1}^{*}=\frac{\left(1-\alpha \right)a+\left(1-\alpha^{2}\right)w_{1}^{*}+\left[1-\alpha \beta -\left(1-\alpha^{2}\right)s\right]e_{1}^{*}+\left(\beta -\alpha \right)e_{2}^{*}}{2\left(1-\alpha^{2}\right)}$$

(4) $$p_{2}^{*}=\frac{\left(1-\alpha \right)a+\left(1-\alpha^{2}\right)w_{2}^{*}+\left(\beta -\alpha \right)e_{1}^{*}+\left(1-\alpha \beta \right)e_{2}^{*}}{2\left(1-\alpha^{2}\right)}$$

Based on the reaction functions, if the manufacturer produces and sells complementary products simultaneously, the retail price of product 1 positively relates only to the product’s wholesale price. In other words, the retail price is independent of the wholesale price of the complementary product 2, and relates positively to the greenness of both products under the wholesale price contract.

The manufacturer makes optimal wholesale price and product greenness decisions, based on perfectly rational expectations of the retailer’s response function. Substituting $p_{1}\left(w_{1}^{*},w_{2}^{*},e_{1}^{*},e_{2}^{*}\right)$ and $p_{2}\left(w_{1}^{*},w_{2}^{*},e_{1}^{*},e_{2}^{*}\right)$ into $\Pi_{m}$ and solving $\frac{\partial \Pi_{m}}{\partial w_{1}^{*}}=0$, $\frac{\partial \Pi_{m}}{\partial w_{2}^{*}}=0$, $\frac{\partial \Pi_{m}}{\partial e_{1}^{*}}=0$, and $\frac{\partial \Pi_{m}}{\partial e_{2}^{*}}=0$, we can obtain the results presented in proposition 2.

**Proposition** **2.** *Under the wholesale price contract, the manufacturer’s optimal decisions are as follows*:

(5) $$e_{1}^{*}=\frac{\begin{array}{c} \left[4\eta \left(1-\alpha^{2}\right)-\left(1+\beta^{2}\right)+2\alpha \beta \right]\left[1+\beta +\left(1+\alpha \right)s\right]\left(1-\alpha \right)a+\\ \left[2\beta -\alpha \left(1+\beta^{2}\right)+\left(1-\alpha^{2}\right)\beta s\right]\left(1+\beta \right)\left(1-\alpha \right)a \end{array}}{\begin{array}{c} \left[4\eta \left(1-\alpha^{2}\right)-\left(1+s\right)\left(\left(1-\alpha \beta \right)+\left(1-\alpha^{2}\right)s-\left(\beta +\alpha s\right)\left(\beta -\alpha \right)\right)\right]\left[4\eta \left(1-\alpha^{2}\right)-\left(1+\beta^{2}\right)+2\alpha \beta \right]-\\ \left[\beta \left(1-\alpha \beta \right)+\beta \left(1-\alpha^{2}\right)s+\beta -\alpha \right]\left[2\beta -\alpha \left(1+\beta^{2}\right)+\left(1-\alpha^{2}\right)\beta s\right] \end{array}}\;$$

(6) $$e_{2}^{*}=\frac{\begin{array}{c} \left[4\eta \left(1-\alpha^{2}\right)-\left(1+s\right)\left(\left(1-\alpha \beta \right)+\left(1-\alpha^{2}\right)s-\left(\beta +\alpha s\right)\left(\beta -\alpha \right)\right)\right]\left(1+\beta \right)(1-\alpha )a+\\ \left[\left(1+\beta \right)\left(1-\alpha \beta \right)+\beta \left(1-\alpha^{2}\right)s\right][1+\beta +\left(1+\alpha \right)s]\left(1-\alpha \right)a \end{array}}{\begin{array}{c} \left[4\eta \left(1-\alpha^{2}\right)-\left(1+s\right)\left(\left(1-\alpha \beta \right)+\left(1-\alpha^{2}\right)s-\left(\beta +\alpha s\right)\left(\beta -\alpha \right)\right)\right]\left[4\eta \left(1-\alpha^{2}\right)-\left(1+\beta^{2}\right)+2\alpha \beta \right]-\\ \left[\beta \left(1-\alpha \beta \right)+\beta \left(1-\alpha^{2}\right)s+\beta -\alpha \right]\left[2\beta -\alpha \left(1+\beta^{2}\right)+\left(1-\alpha^{2}\right)\beta s\right] \end{array}}$$

(7) $$w_{1}^{*}=\frac{\left(1-\alpha \right)a+\left[\left(1-\alpha \beta \right)+\left(1-\alpha^{2}\right)s\right]e_{1}^{*}+\left(\beta -\alpha \right)e_{2}^{*}}{2\left(1-\alpha^{2}\right)}\;$$

(8) $$w_{2}^{*}=\frac{\left(1-\alpha \right)a+\left(\beta -\alpha \right)e_{1}^{*}+\left(1-\alpha \beta \right)e_{2}^{*}}{2\left(1-\alpha^{2}\right)}$$

According to the optimal decisions in proposition 2, the manufacturer’s wholesale prices for two complementary products relate not only to the cross-price elasticity and green spillover effect, but also relate positively to their greenness. It implies that the greenness of product 2 affects the wholesale price of both itself and product 1. Similarly, by comparing and derivatizing the optimal decisions ($e_{1}^{*}$, $e_{2}^{*}$, $w_{1}^{*}$, $w_{2}^{*}$, $p_{1}^{*}$, $p_{2}^{*}$), corollary 2 can be obtained as follows:

**Corollary** **2.** *Under the wholesale price contract, in relation to the greenness of the complementary products: when* s>0, $e_{1}^{*}>e_{2}^{*}$; the relationship between the government green subsidy rate and product greenness is $\frac{\partial e_{1}^{*}}{\partial s}>0$, $\frac{\partial e_{2}^{*}}{\partial s}>0$, $\frac{\partial e_{1}^{*}}{\partial s}>\frac{\partial e_{2}^{*}}{\partial s}$*, and the relationship between the green subsidy rate and prices is* $\frac{\partial p_{1}^{*}}{\partial s}>0$, $\frac{\partial p_{2}^{*}}{\partial s}>0$, $\frac{\partial w_{1}^{*}}{\partial s}>0$, $\frac{\partial w_{2}^{*}}{\partial s}>0$.

Regarding corollary 2, under the wholesale price contract, the greenness of product 1 is greater than that of its complementary product 2, because of the donation of the government’s green subsidy to product 1. This difference implies that the manufacturers tend to make their manufacturing process greener with subsidized products. However, as the government increases the green subsidy rate for product 1, the manufacturer increases the optimal greenness of both complementary products. The only exception is the greenness incentive effect of the green subsidy on the directly subsidized product, which is greater than its green innovation incentive effect on the other complementary products, because the spillover effect on the product demand is less than 1, β<1. Therefore, when the government considers green product subsidies, if the relevance of complementary products can be perceived as one of the influencing factors, the promotion effect of subsidies achieves double the amount with half the effort. For example, the effect of a green subsidy for one car retailer brand that provides automobiles and complementary products from the same manufacture is better than that for multiple competing brands of household appliances. Finally, according to the third part of corollary 2, under the wholesale price contract, the wholesale prices and the retailer prices increase with the greenness subsidy rate.

The supply chain profit ($\Pi_{sc}^{*}=\Pi_{m}^{*}+\Pi_{r}^{*}$) is calculated according to the optimal decisions of the supply chain members, and corollary 3 is obtained by comparing it with the centralized model.

**Corollary** **3.** *Under the wholesale price contract,* $e_{1}^{*}<e_{1}^{c*}$, $e_{2}^{*}<e_{2}^{c*}$, and$\Pi_{sc}^{*}<\Pi_{sc}^{c*}$.

According to corollary 3, under the wholesale price contract, the double marginal utility affects the optimal decision and profit due to the decentralized decision-making process among supply chain members with their own profit maximization as the decision objective. In this case, the greenness of the products and the overall profit of the supply chain are lower than those of the decision-making process in the centralized model. Therefore, the next attempt is to optimize and coordinate the supply chain through the sharing contracts.

Under the subsidy-sharing contract (denoted by the superscript *t*), the retailer shares a certain percentage of the government’s green subsidy to motivate the manufacturer to improve the greenness of the product. According to the assumptions, the model can be constructed as follows:

(9) $$\underset{w_{1}^{t},w_{2}^{t},e_{1}^{t},e_{2}^{t}}{\max\Pi_{m}^{t}}=[w_{1}^{t}+(1-t)se_{1}^{t}]D_{1}+w_{2}^{t}D_{2}-\frac{1}{2}\eta e_{1}^{t2}-\frac{1}{2}\eta e_{2}^{t2}$$

(10) $$s.t.\underset{p_{1}^{t},p_{2}^{t}}{\max\Pi_{r}^{t}}=(p_{1}^{t}-w_{1}^{t}+tse_{1}^{t})D_{1}+(p_{2}^{t}-w_{2}^{t})D_{2}$$

Solving this in reverse order, the following proposition 3 can be obtained:

**Proposition** **3.** *Under the subsidy-sharing contract, the manufacturer’s optimal equilibrium decisions are:*

$e_{1}^{t*}=e_{1}^{*}$, $e_{2}^{t*}=e_{2}^{*}$, $w_{2}^{t*}=w_{2}^{*}$, *and*$w_{1}^{t*}=\frac{\left(1-\alpha \right)a+\left[\left(1-\alpha \beta \right)-\left(1-\alpha^{2}\right)\left(1-2t\right)s\right]e_{1}^{t*}+\left(\beta -\alpha \right)e_{2}^{t*}}{2\left(1-\alpha^{2}\right)}$.

*The optimal response functions of the retailer are as follows:*

(11) $$p_{1}^{t*}=\frac{\left(1-\alpha \right)a+\left(1-\alpha^{2}\right)w_{1}^{t*}+\left[\left(1-\alpha \beta \right)-\left(1-\alpha^{2}\right)ts\right]e_{1}^{t*}+\left(\beta -\alpha \right)e_{2}^{t*}}{2\left(1-\alpha^{2}\right)}$$

(12) $$p_{2}^{t*}=\frac{\left(1-\alpha \right)a+\left(1-\alpha^{2}\right)w_{2}^{t*}+\left(\beta -\alpha \right)e_{1}^{t*}+\left(1-\alpha \beta \right)e_{2}^{t*}}{2\left(1-\alpha^{2}\right)}\;$$

The wholesale price of product 1 under the green subsidy-sharing contract can be rewritten as $w_{1}^{t*}=\frac{\left(1-\alpha \right)a+\left(1-\alpha \beta \right)e_{1}^{t*}-\left(1-\alpha^{2}\right)se_{1}^{t*}+\left(\beta -\alpha \right)e_{2}^{t*}}{2\left(1-\alpha^{2}\right)}+se_{1}^{t*}t=w_{1}^{t*-}+se_{1}^{t*}t$, then there is $\frac{\partial w_{1}^{t*}}{\partial t}$ > 0, where the wholesale price increases with the green subsidy ratio of the retailer. In other words, the manufacture will decrease the wholesale price for retailer, when he shares the green subsidies. By rearranging the retail price further, we can obtain $p_{1}^{t*}=\frac{\left(1-\alpha \right)a+\left(1-\alpha^{2}\right)w_{1}^{t*-}+\left(1-\alpha \beta \right)e_{1}^{t*}+\left(\beta -\alpha \right)e_{2}^{t*}}{2\left(1-\alpha^{2}\right)}$, which has no significance in relation to the sharing proportion, t. Then, according to the demand function of the products in the above equation, it can also be inferred that the sharing proportion does not affect the market demand and supply chain profit. Therefore, corollary 4 is obtained as follows:

**Corollary** **4.** *In comparison to the wholesale price contract, the green subsidy-sharing contract decreases the manufacture’s wholesale price of product 1, while it does not provide manufacturers with incentives to increase the greenness of their products, and is ineffective in supply chain optimization*.

### 4.3. Revenue-Sharing Contracts

Under the revenue-sharing contract (marked by the superscript λ), the manufacturer and the retailer share the revenue from the sale of product 1, where the retailer shares the revenue in the proportion λ, and the manufacturer shares the remaining (1−λ), assuming that the allocation proportion is first decided through negotiation or other means before the pricing and green innovation game. Then, the profits of the manufacturer and retailer with a government green subsidy can be expressed separately as:

(13) $$\Pi_{m}^{\lambda }=w_{1}D_{1}+w_{2}D_{2}+\left(1-\lambda \right)\left(p_{1}-w_{1}+se_{1}\right)D_{1}-\frac{1}{2}\eta e_{1}^{2}-\frac{1}{2}\eta e_{2}^{2}$$

(14) $$\Pi_{r}=\lambda \left(p_{1}-w_{1}+se_{1}\right)D_{1}+\left(p_{2}-w_{2}\right)D_{2}$$

Similar to the wholesale price contract, the optimal response function of the retailer in the revenue-sharing contract case can be obtained, firstly, as:

(15) $$p_{1}^{\lambda *}=\frac{\begin{array}{c} \left[2\lambda -\left(1+\lambda \right)\alpha \right]a+\left[2\lambda -\left(1+\lambda \right)\lambda \alpha^{2}\right]w_{1}^{\lambda *}+\left[2\alpha -\left(1+\lambda \right)\alpha \right]w_{2}^{\lambda *}+\\ \left[2\lambda \left(1-s\right)-\alpha \left(1+\lambda \right)\left(\beta -\lambda \alpha s\right)\right]e_{1}^{\lambda *}+\left[2\lambda \beta -\left(1+\lambda \right)\alpha \right]e_{2}^{\lambda *} \end{array}}{4\lambda -\left(1+\lambda^{2}\right)\alpha^{2}}\;$$

(16) $$p_{2}^{\lambda *}=\frac{\begin{array}{c} \left[2\lambda -\left(1+\lambda \right)\alpha \right]a+\left[2\lambda^{2}\alpha -\left(1+\lambda \right)\lambda \alpha \right]w_{1}^{\lambda *}+\left[2\lambda -\left(1+\lambda \right)\alpha^{2}\right]w_{2}^{\lambda *}+\\ \left[2\lambda \left(\beta -\lambda \alpha s\right)-\alpha \left(1+\lambda \right)\lambda \left(1-s\right)\right]e_{1}^{\lambda *}+\left[2\lambda -\left(1+\lambda \right)\lambda \alpha \beta \right]e_{2}^{\lambda *} \end{array}}{4\lambda -\left(1+\lambda^{2}\right)\alpha^{2}}\;$$

In the retailer’s response function, if $e_{1}^{\lambda *}$ and $e_{2}^{\lambda *}$ are assumed to be the determined product greenness, then the effect of the government subsidy (s) on the retail price is closely related to the value of $\frac{\left[2\lambda \left(1-s\right)-\alpha \left(1+\lambda \right)\left(\beta -\lambda \alpha s\right)\right]e_{1}^{\lambda *}}{4\lambda -\left(1+\lambda^{2}\right)\alpha^{2}}$. Then, the government subsidy coefficient becomes (1+λ)λs, while it is s in the wholesale price contract case. Since $\frac{\partial \left(1+\lambda \right)\lambda }{\partial \lambda }=2\lambda +1>0$, (1+λ)λ becomes smaller as λ decreases, that is, the actual government subsidy coefficient under the revenue-sharing contract decreases. In other words, the fewer the retailer shares from the revenue from product 1, the smaller the actual government subsidy coefficient. Furthermore, when λ=1, the revenue-sharing contract is just equivalent to the wholesale price contract with $p_{1}^{\lambda *}=p_{1}^{*}$. Therefore, under the revenue-sharing contract, when 0<λ<1, the effect of the government green subsidy rate on the retailer’s profit per unit of product becomes smaller, because the government’s subsidy on the greenness is removed. The retailer actually shares the government’s green subsidies with the upstream manufacturer in the form of revenue sharing, transferring a portion of the green subsidies to them. The benefit of this sharing by the retailer encourages the manufacturer to increase the product greenness, and thus increase the actual subsidy amount and profit. Under the revenue-sharing contract, unlike the wholesale price contract, the retailer’s product sale price relates positively to the wholesale price and greenness of not only the product itself, but also the complementary product.

The manufacturer makes optimal decisions based on the retailer’s response function, and obtains the optimal wholesale prices and product greenness under the revenue-sharing contract, as in proposition 4. We included the expressions of capital letters in Appendix A

**Proposition** **4.** *Under a revenue-sharing contract, the manufacturer’s optimal wholesale price and product greenness decisions are as follows*:

(17) $$e_{1}^{\lambda *}=\frac{\begin{array}{l} \left[A_{4}(B_{2}D_{1}-B_{1}D_{2})+B_{4}(A_{1}D_{2}-A_{2}D_{1})+C_{4}(A_{2}B_{1}-A_{2}B_{1})\right]\left[(A_{2}B_{1}-A_{1}B_{2})E_{3}+(B_{3}A_{1}-A_{3}B_{1})E_{2}+(A_{3}B_{2}-B_{3}A_{2})E_{1}\right]\\ -[A_{3}(B_{2}D_{1}-B_{1}D_{2})+B_{3}(A_{1}D_{2}-A_{2}D_{1})+C_{3}(A_{2}B_{1}-A_{2}B_{1})]\left[(A_{2}B_{1}-A_{1}B_{2})E_{4}+(B_{4}A_{1}-A_{4}B_{1})E_{2}+(A_{4}B_{2}-B_{4}A_{2})E_{1}\right] \end{array}}{\begin{array}{l} \left[A_{3}(B_{2}C_{1}-B_{1}C_{2})+B_{3}(A_{1}C_{2}-A_{2}C_{1})+C_{3}(A_{2}B_{1}-A_{2}B_{1})\right]\left[A_{4}(B_{2}D_{1}-B_{1}D_{2})+B_{4}(A_{1}D_{2}-A_{2}D_{1})+C_{4}(A_{2}B_{1}-A_{2}B_{1})\right]\\ -[A_{4}(B_{2}C_{1}-B_{1}C_{2})+B_{4}(A_{1}C_{2}-A_{2}C_{1})+C_{4}(A_{2}B_{1}-A_{2}B_{1})]\left[A_{3}(B_{2}D_{1}-B_{1}D_{2})+B_{3}(A_{1}D_{2}-A_{2}D_{1})+C_{3}(A_{2}B_{1}-A_{2}B_{1})\right] \end{array}}$$

(18) $$e_{2}^{\lambda *}=\frac{\begin{array}{l} \left[A_{3}(B_{2}C_{1}-B_{1}C_{2})+B_{3}(A_{1}C_{2}-A_{2}C_{1})+C_{3}(A_{2}B_{1}-A_{2}B_{1})\right]\left[(A_{2}B_{1}-A_{1}B_{2})E_{4}+(B_{4}A_{1}-A_{4}B_{1})E_{2}+(A_{4}B_{2}-B_{4}A_{2})E_{1}\right]\\ -[A_{4}(B_{2}C_{1}-B_{1}C_{2})+B_{4}(A_{1}C_{2}-A_{2}C_{1})+C_{4}(A_{2}B_{1}-A_{2}B_{1})]\left[(A_{2}B_{1}-A_{1}B_{2})E_{3}+(B_{3}A_{1}-A_{3}B_{1})E_{2}+(A_{3}B_{2}-B_{3}A_{2})E_{1}\right] \end{array}}{\begin{array}{l} \left[A_{3}(B_{2}C_{1}-B_{1}C_{2})+B_{3}(A_{1}C_{2}-A_{2}C_{1})+C_{3}(A_{2}B_{1}-A_{2}B_{1})\right]\left[A_{4}(B_{2}D_{1}-B_{1}D_{2})+B_{4}(A_{1}D_{2}-A_{2}D_{1})+C_{4}(A_{2}B_{1}-A_{2}B_{1})\right]\\ -[A_{4}(B_{2}C_{1}-B_{1}C_{2})+B_{4}(A_{1}C_{2}-A_{2}C_{1})+C_{4}(A_{2}B_{1}-A_{2}B_{1})]\left[A_{3}(B_{2}D_{1}-B_{1}D_{2})+B_{3}(A_{1}D_{2}-A_{2}D_{1})+C_{3}(A_{2}B_{1}-A_{2}B_{1})\right] \end{array}}$$

(19) $$w_{1}^{\lambda *}=\frac{B_{1}E_{2}-B_{2}E_{1}+\left(B_{2}C_{1}-B_{1}C_{2}\right)e_{1}^{\lambda *}+\left(B_{2}D_{1}-B_{1}D_{2}\right)e_{2}^{\lambda *}}{A_{2}B_{1}-A_{1}B_{2}}\;$$

(20) $$w_{2}^{\lambda *}=\frac{A_{2}E_{1}-A_{1}E_{2}+\left(A_{1}C_{2}-A_{2}C_{1}\right)e_{1}^{\lambda *}+\left(A_{1}D_{2}-A_{2}D_{1}\right)e_{2}^{\lambda *}}{A_{2}B_{1}-A_{1}B_{2}}\;$$

Based on the optimal decision-making process for the product greenness in proposition 4, we can obtain corollary 5 from the comparisons with the wholesale price contract and centralized decision cases.

**Corollary** **5.** *Under the revenue-sharing contract, the greenness of the complementary products are* $e_{1}^{\lambda *}>e_{2}^{\lambda *}$; the influence of the government green subsidy coefficients on the greenness of the product are$\frac{\partial e_{1}^{\lambda *}}{\partial s}>0$*,*$\;\frac{\partial e_{2}^{\lambda *}}{\partial s}>0$*, and*$\frac{\partial e_{1}^{\lambda *}}{\partial s}>\frac{\partial e_{1}^{*}}{\partial s}$*,*$\frac{\partial e_{2}^{\lambda *}}{\partial s}>\frac{\partial e_{2}^{*}}{\partial s}$*and, compared with the centralized decision situation and the wholesale price contract, we can determine that*$e_{1}^{*}<e_{1}^{\lambda *}<e_{1}^{c*}$,$e_{2}^{\lambda *}<e_{2}^{*}<e_{2}^{c*}$,$\left(e_{1}^{*}+e_{2}^{*}\right)<\left(e_{1}^{\lambda *}+e_{2}^{\lambda *}\right)$.

The above corollary is obtained by analyzing the coefficients of s in the game-solving process. For example, the coefficient, χ, in $C_{1}$ with respect to s is a decreasing function of λ, that is, when λ decreases, χ increases, so s has a significant effect on the product greenness. When λ=1, it happens to be the greenness decision in the case of the wholesale price contract, so when 0<λ<1, the product is greener under the revenue-sharing contract than that under the wholesale price contract. The other analyses are similar.

Based on corollary 5, the revenue-sharing contract between the manufacturer and retailer of the complementary product supply chain, in the case of the government’s green product subsidies, incentivizes the manufacturer to increase the green innovation and greenness of product 1. However, it still does not achieve the incentive effect under the centralized decision model. Under the revenue-sharing contract, the greenness of complementary product 2 is less than that under the wholesale price contract. Nevertheless, the total greenness of both products is still greater than that under the wholesale price contract.

With the optimal decisions of the manufacturer and retailer, the optimal profit of the manufacturer is $\Pi_{s}^{\lambda *}=w_{1}^{\lambda *}D_{1}^{\lambda *}+w_{2}^{\lambda *}D_{2}^{\lambda *}+\left(1-\lambda \right)\left(p_{1}^{\lambda *}-w_{1}^{\lambda *}+se_{1}^{\lambda *}\right)D_{1}^{\lambda *}-\frac{1}{2}\eta e_{1}^{\lambda *2}-\frac{1}{2}\eta e_{2}^{\lambda *2}$. When λ=0, there is exactly $\Pi_{s1}^{\lambda *}\left(\lambda =0\right)=\left[w_{1}^{\lambda *}D_{1}^{\lambda *}+\left(1-\lambda \right)\left(p_{1}^{\lambda *}-w_{1}^{\lambda *}+se_{1}^{\lambda *}\right)D_{1}^{\lambda *}-\frac{1}{2}\eta e_{1}^{\lambda *2}\right]=\Pi_{s1}^{c*}$; the manufacturer’s profit from product 1 is equal to the maximum profit of the centralized model and the overall profit of the supply chain for product 1 is also maximized, i.e., coordination is achieved. However, in this case, the retailer gains zero profit from selling product 1, and then the retailer rejects this revenue-sharing contract. Therefore, the revenue-sharing contract is acceptable only if 0<λ<1 and λ is the appropriate value, and then $\Pi_{s1}^{\lambda *}(0<\lambda <1)<\Pi_{s1}^{c*}$, $\Pi_{s2}^{\lambda *}(0<\lambda <1)<\Pi_{s2}^{c*}$.

Solving $\frac{\partial \Pi_{r}^{\lambda *}}{\partial \lambda }=0$ attains the optimal $\lambda_{r}^{*}$, with which the retailer earns the highest profit under the revenue-sharing contract. If λ=1, then $\Pi_{s1}^{\lambda *}\left(\lambda =1\right)=\Pi_{s1}^{*}$ and $\Pi_{s2}^{\lambda *}\left(\lambda =1\right)=\Pi_{s2}^{*}$, i.e., the manufacturer’s revenue from products 1 and 2 is just equal to the revenue under the wholesale price contract. According to corollary 4, when the value of the revenue-sharing ratio decreases from 1 to $\lambda_{r}^{*}$, then $\Pi_{s}^{\lambda *}$ increases and $\Pi_{s}^{\lambda *}>\Pi_{s}^{*}$. Therefore, corollary 6 is presented as follows:

**Corollary** **6.** *In the case of a government green subsidy, when the revenue-sharing ratio is appropriate, then there are* $\Pi_{sc}^{*}<\Pi_{sc}^{\lambda *}<\Pi_{sc}^{c*}$,$\Pi_{s}^{*}<\Pi_{s}^{\lambda *}$, and$\Pi_{r}^{*}<\Pi_{r}^{\lambda *}$.

According to corollary 6, the revenue-sharing contract for product 1 cannot achieve coordination in the supply chain of complementary products, but when the sharing ratio is appropriately valued, it can result in the profit of all supply chain members achieving a Pareto improvement.

### 4.4. Cost-Sharing Contracts

Another method of cooperation among supply chain members is innovation cost-sharing, in which the retailer shares the green manufacturing cost of the manufacturer’s green product. Under the cost-sharing contract (denoted by the superscript k), the retailer promises to share the manufacturer’s green manufacturing cost according to sharing ratio k. The manufacturer accepts the contract, then decides the wholesale prices and the product greenness, and, finally, the retailer decides the prices of the products and offers the complementary products to the consumers. Given the assumptions and the retailer’s sharing ratio, k, the price game process is modeled as follows:

(21) $$\underset{w_{1}^{k},w_{2}^{k},e_{1}^{k},e_{2}^{k}}{\max\Pi_{m}^{k}}=[w_{1}^{k}+se_{1}^{k}]D_{1}+w_{2}^{k}D_{2}-(1-k)\frac{1}{2}\eta e_{1}^{k2}-\frac{1}{2}\eta e_{2}^{k2}\;$$

(22) $$s.t.\underset{p_{1}^{k},p_{2}^{k}}{\max\Pi_{r}^{k}}=(p_{1}^{k}-w_{1}^{k})D_{1}+(p_{2}^{k}-w_{2}^{k})D_{2}-k\frac{1}{2}\eta e_{1}^{k2}$$

By solving in reverse order, proposition 5 can be obtained.

**Proposition** **5.** *Under the cost-sharing contract and given the retailer’s cost-sharing ratio, the manufacturer’s optimal equilibrium decisions are as follows*:

(23) $$e_{1}^{*}\left(k\right)=\frac{\begin{array}{c} \left[4\eta \left(1-\alpha^{2}\right)-\left(1+\beta^{2}\right)+2\alpha \beta \right][1+\beta +\left(1+\alpha \right)s]\left(1-\alpha \right)a+\\ \left[2\beta -\alpha \left(1+\beta^{2}\right)+\left(1-\alpha^{2}\right)\beta s\right]\left(1+\beta \right)\left(1-\alpha \right)a \end{array}}{\begin{array}{l} {}[4\left(1-\alpha^{2}\right)\left(1-k\right)\eta -\left(\beta +\alpha s\right)\left(\beta -\alpha \right)-\left(1+s\right)\left(1+s-\alpha \beta -\alpha^{2}s\right)]\left[4\eta \left(1-\alpha^{2}\right)-\left(1+\beta^{2}\right)+2\alpha \beta \right]\\ -\left[\beta \left(1-\alpha \beta \right)+\beta \left(1-\alpha^{2}\right)s+\beta -\alpha \right]\left[2\beta -\alpha \left(1+\beta^{2}\right)+\left(1-\alpha^{2}\right)\beta s\right] \end{array}}$$

(24) $$e_{2}^{*}\left(k\right)=\frac{\begin{array}{c} \left[4\left(1-\alpha^{2}\right)\left(1-k\right)\eta -\left(\beta +\alpha s\right)\left(\beta -\alpha \right)-\left(1+s\right)\left(1+s-\alpha \beta -\alpha^{2}s\right)\right](1+\beta )(1-\alpha )a+\\ \left[(1+\beta )(1-\alpha \beta )+\beta \left(1-\alpha^{2}\right)s\right][1+\beta +\left(1+\alpha \right)s]\left(1-\alpha \right)a \end{array}}{\begin{array}{l} {}[4\left(1-\alpha^{2}\right)\left(1-k\right)\eta -\left(\beta +\alpha s\right)\left(\beta -\alpha \right)-\left(1+s\right)\left(1+s-\alpha \beta -\alpha^{2}s\right)]\left[4\eta \left(1-\alpha^{2}\right)-\left(1+\beta^{2}\right)+2\alpha \beta \right]\\ -\left[\beta \left(1-\alpha \beta \right)+\beta \left(1-\alpha^{2}\right)s+\beta -\alpha \right]\left[2\beta -\alpha \left(1+\beta^{2}\right)+\left(1-\alpha^{2}\right)\beta s\right] \end{array}}$$

(25) $$w_{1}^{k*}\left(k\right)=\frac{\left(1-\alpha \right)a+\left[\left(1-\alpha \beta \right)+\left(1-\alpha^{2}\right)s\right]e_{1}^{k*}+\left(\beta -\alpha \right)e_{2}^{k*}}{2\left(1-\alpha^{2}\right)}\;$$

(26) $$w_{2}^{k*}\left(k\right)=\frac{\left(1-\alpha \right)a+\left(\beta -\alpha \right)e_{1}^{k*}+\left(1-\alpha \beta \right)e_{2}^{k*}}{2\left(1-\alpha^{2}\right)}\;$$

The optimal response functions of the retailer are below.

(27) $$p_{1}^{k*}\left(k\right)=\frac{\left(1-\alpha \right)a+\left(1-\alpha^{2}\right)w_{1}^{k*}+\left[\left(1-\alpha \beta \right)-\left(1-\alpha^{2}\right)s\right]e_{1}^{k*}+\left(\beta -\alpha \right)e_{2}^{k*}}{2\left(1-\alpha^{2}\right)}\;$$

(28) $$p_{2}^{k*}\left(k\right)=\frac{\left(1-\alpha \right)a+\left(1-\alpha^{2}\right)w_{2}^{k*}+\left(\beta -\alpha \right)e_{1}^{k*}+\left(1-\alpha \beta \right)e_{2}^{k*}}{2\left(1-\alpha^{2}\right)}\;$$

According to the optimal solution in the price game, we can obtain and compare the derivation of the optimal greenness of products with respect to s and k to obtain corollary 7.

**Corollary** **7.** *Under the cost-sharing contract, when* k*is appropriate,* $e_{1}^{k*}>e_{2}^{k*}$. The influence of the government green subsidy coefficient on the greenness of the products is$\;\frac{\partial e_{1}^{k*}}{\partial s}>0$ *and* $\frac{\partial e_{2}^{k*}}{\partial s}>0$*, and there are* $\frac{\partial e_{1}^{k*}}{\partial s}>\frac{\partial e_{2}^{k*}}{\partial s}$, $\frac{\partial e_{1}^{\lambda *}}{\partial s}>\frac{\partial e_{1}^{*}}{\partial s}$, $\frac{\partial e_{2}^{\lambda *}}{\partial s}>\frac{\partial e_{2}^{*}}{\partial s}$*. Compared with the centralized decision situation and the wholesale price contract,* $e_{1}^{*}<e_{1}^{k*}<e_{1}^{c*}$and$e_{2}^{*}<e_{2}^{k*}<e_{2}^{c*}$.

Similar to the revenue-sharing contract, under the cost-sharing contract and if the sharing ratio is appropriate, the government green subsidies incentivize manufacturers to increase the greenness of product 1 and complementary product 2, while the manufacturer is inclined to increase the greenness of subsidized product 1. Compared to the wholesale price contract, the incentive of the government’s green subsidies is more encouraging under the cost-sharing contract, and the greenness of products 1 and 2 is greater than that in the case of the wholesale price contract, while it is lower than that in the concentration decision case.

Based on the original contracting process, the retailer decides its green cost-share ratio, $k_{r}^{*}$, based on its own profit maximization. The solution model is as follows:

(29) $$\begin{array}{l} \underset{k_{r}^{*}}{\max\Pi_{r}^{k*}}(w_{1}^{k}(k_{r}^{*}),w_{2}^{k}(k_{r}^{*}),e_{1}^{k}(k_{r}^{*}),e_{2}^{k}(k_{r}^{*}))\\ s.t.\Pi_{m}^{k*}(w_{1}^{k}(k_{r}^{*}),w_{2}^{k}(k_{r}^{*}),e_{1}^{k}(k_{r}^{*}),e_{2}^{k}(k_{r}^{*}))>\Pi_{r}^{*}\\ \Pi_{m}^{k*}(w_{1}^{k}(k_{r}^{*}),w_{2}^{k}(k_{r}^{*}),e_{1}^{k}(k_{r}^{*}),e_{2}^{k}(k_{r}^{*}))>\Pi_{m}^{*} \end{array}$$

When k=0, the decisions and profits of the supply chain members are precisely the same as the wholesale price contract. The optimal decision and profit functions can be organized to prove that the first- and second-order conditions for the optimal solution exist for $max\pi_{r}^{k*}\left[w_{1}^{k}\left(k_{r}^{*}\right),w_{2}^{k}\left(k_{r}^{*}\right),e_{1}^{k}\left(k_{r}^{*}\right),e_{2}^{k}\left(k_{r}^{*}\right)\right]$, and then there exists an optimal solution, $k_{r}^{k*}$. Thus, there is corollary 8.

**Corollary** **8.** *Under a cost-sharing contract, the profits of the supply chain members are consistent with the following statements:* $\Pi_{sc}^{*}<\Pi_{sc}^{k*}<\Pi_{sc}^{c*}$,$\Pi_{s}^{*}<\Pi_{s}^{k*}$, and$\Pi_{r}^{*}<\Pi_{r}^{k*}$.

The cost-sharing contract increases the profits of manufacturers, retailers, and the supply chain, when the sharing ratio is appropriate. However, in a complementary product supply chain, cost-sharing contracts do not enable the coordination of the supply chain.

## 5. Numerical Simulations

This section analyzes the effects of complementary product correlations (cross-price elasticity and green spillover effects) on supply chain members’ decisions, profits, and contracts using appropriate parameter assignments. In addition, the optimization effects of the supply chain contracts on the supply chain, as mentioned above, are compared and investigated. We designed the parameter values used in this paper by learning the related research, such as Dehghanbaghi and Mohsen (2017) [12], Jamali and Morteza (2019) [38], Samar et al. (2011) [39], Ma et al. (2017) [40] and Hamed et al. (2020) [41], and present the data in Appendix B.

**Figure 2:**
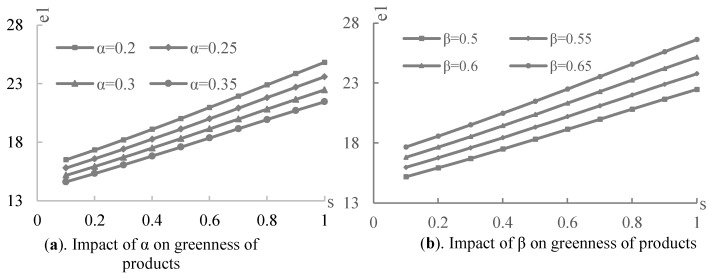
The impact of the complementary product correlations.

Firstly, in the centralized decision model, to be more realistic as well as consistent with the model assumptions, we set the initial parameter values as follows: a=100, η=5, s∈[0.1,1]. Then, the effect of the cross-price elasticity coefficient (α=0.2,0.25,0.3,0.35) and the green spillover coefficient (β=0.5,0.55,0.6,0.65) of the complementary products on the greenness of the supply chain products were examined, as shown in Figure 2a,b, respectively. From the figures, the greenness of the products in the supply chain increases with the government green subsidy rate (consistent with corollary 1 in the present paper). Nonetheless, the cross-price elasticity and the green spillover coefficient of the complementary products have different effects on the greenness of product 1. In Figure 2a, for the same government subsidy rate, the greenness of product 1 decreases with the cross-price elasticity coefficient. In Figure 2b, the higher the green innovation spillover coefficient of the complementary product, the higher the greenness of product 1. However, these complementary characteristics have the same effect on the greenness of complementary product 2.

**Figure 3:**
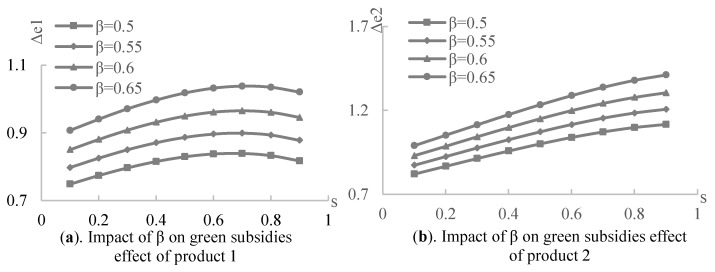
The impact of the green spillover effect.

Therefore, considering the green innovation of the complementary product supply chain, attention needs to be paid to the mutual influence of the complementary products. Moreover, the green spillover effect can be fully utilized, for example, by consciously increasing the green innovation spillover effect among products through product design and promotion. This spillover effect increases the greenness and profit of the overall product group. As shown in Figure 3, the greenness caused by the government green subsidies increases as the green spillover coefficient increases for both subsidized product 1 and unsubsidized complementary product 2. When β=0.65, the increase in the government green subsidy rate brings the change of greenness ($\Delta e_{1}$,$\Delta e_{2}$) of products 1 and 2 to its highest, as shown in Figure 3a,b.

**Figure 4:**
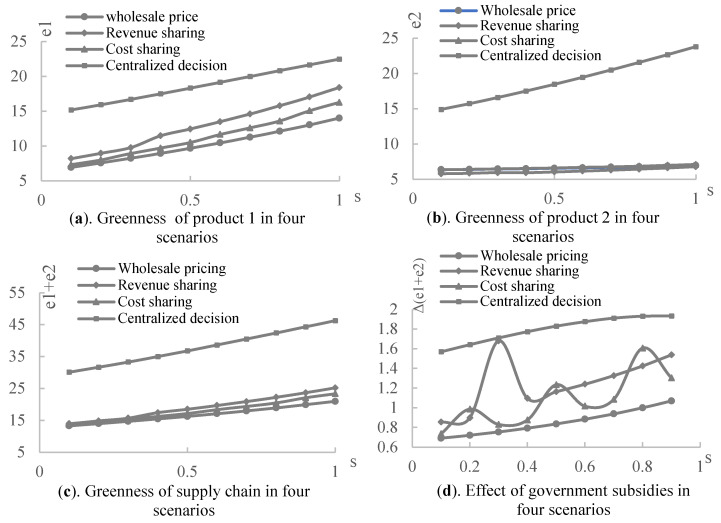
The effect of government subsidies on greenness under four scenarios.

Next, the impact of the government green subsidies on the product greenness under three contracts was experimentally analyzed, assuming that a=100,η=5,α=0.3,β=0.5,s∈[0.1,1], and the sharing apportionment ratios of the retailer in the revenue-sharing contract and cost-sharing contract are the retailer’s optimal decisions made under different subsidy rates. As shown in Figure 4, the greenness of the products and supply chain increases with the government green subsidy rate. It implies that the government’s green subsidy rate constantly improves the greenness of the products and supply chain in all scenarios and contracts. Figure 4a displays that product 1, subsidized by the government, has the highest greenness in the centralized decision model, and its greenness under both the revenue and cost-sharing contract is higher than that under the wholesale price contract. Based on Figure 4b, complementary product 2 also has the highest greenness in the centralized decision case, but its greenness under both the revenue and cost-sharing contracts is lower than that under the wholesale price contract. Therefore, in the cooperative case, the manufacturer is more inclined towards a green production of the subsidized product. Suppose that the greenness of the whole supply chain is the sum of the greenness of product 1 and complementary product 2, then Figure 4c shows that the greenness of the supply chain is highest in the centralized decision-making scenario, followed by the revenue-sharing contract, then the cost-sharing contract. The lowest greenness is evident in the wholesale price contract. Therefore, the double margins produced by the decentralized decision-making process among supply chain members reduce the greenness of the supply chain. Based on the wholesale price contract, however, supply chain members who can cooperate through revenue or cost-sharing continually improve the overall greenness of the supply chain, although it may reduce the greenness of the complementary products. In addition, Figure 4d illustrates that both the revenue- and cost-sharing contracts improve the incentive effect of government green subsidies on green manufacturing in the supply chain to different degrees, according to the supply chain members’ negotiations over the contract parameters.

**Figure 5:**
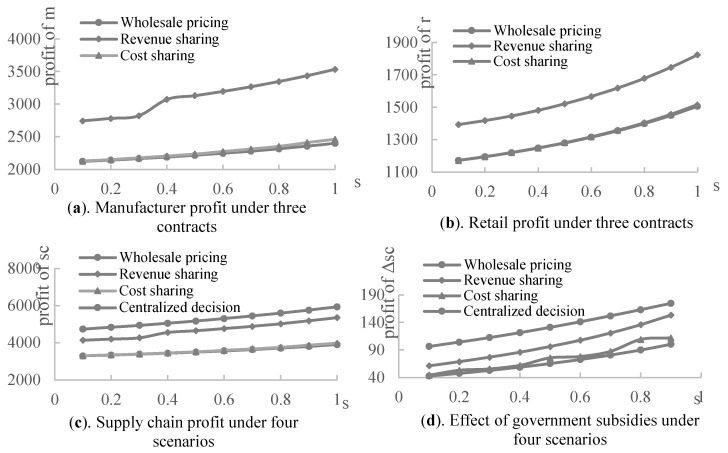
The effect of government subsidies on the supply chain profits.

Figure 5 represents the profits of the supply chain members under three contracts. We can observe that the profits of the manufacturer, retailer, and overall supply chain under the revenue-sharing contract are all significantly higher than those under the wholesale price contract, while the profits of each member under the cost-sharing contract are slightly higher than the wholesale price contract. Figure 5c shows that the supply chain profits under all three contracts are lower than those in the centralized decision-making scenario. Therefore, for the supply chain of the complementary products considering government green subsidies, the revenue- and cost-sharing contracts achieve a Pareto improvement of the supply chain members and the wholesale price contract, but cannot achieve an overall supply chain coordination. Among them, the revenue-sharing contract has the best optimization effect on the supply chain. Based on Figure 5d, the supply chain contract also improves the effect of the government green subsidies.

**Figure 6:**
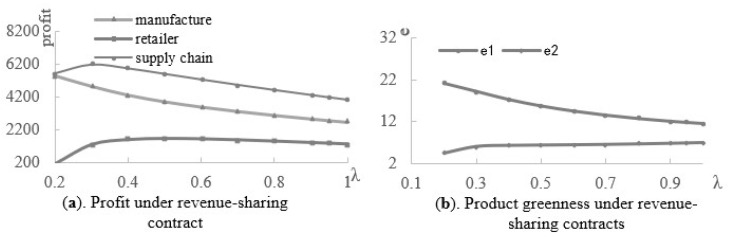
The effect of the revenue-sharing ratio.

**Figure 7:**
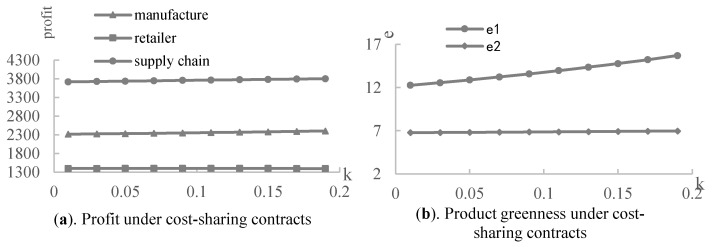
The effect of the cost-sharing ratio.

Then, we employed the numerical experiments to analyze the effect of the retailer’s share ratios under the revenue-sharing and cost-sharing contracts with the following assumptions: a=100,η=5,α=0.3,β=0.5,s=0.8, λ∈[0.2,1], and k∈[0.01,0.19]. Under the revenue-sharing contract, Figure 6a shows that the retailer’s profit firstly increases and then decreases, as his share ratio of the revenue increases. The largest profit is at point $\lambda_{r}^{*}$. The profits of manufacturer and the whole supply chain decrease with the retailer’s revenue share ratio. Therefore, the performance of the manufacturer and the supply chain decline as the share ratio of the retailer increases, although the revenue-sharing contracts improve the performance of the supply chain members and the supply chain as a whole. Regarding Figure 6b, the greenness of product 1 decreases as the share ratio of the retailers increases, while that of product 2 increases slightly. According to Figure 7, under the cost-sharing contract, the greenness and profit of the product in the supply chain increase as the cost-sharing ratio of the retailer increases, and the retailer obtains the largest profit at point $k_{r}^{*}$. 

**Figure 8:**
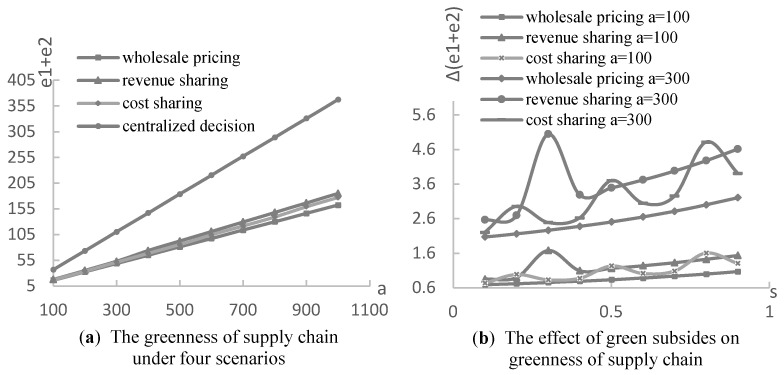
The effect of the consumers with environmental awareness on the greenness of the supply chain.

**Figure 9:**
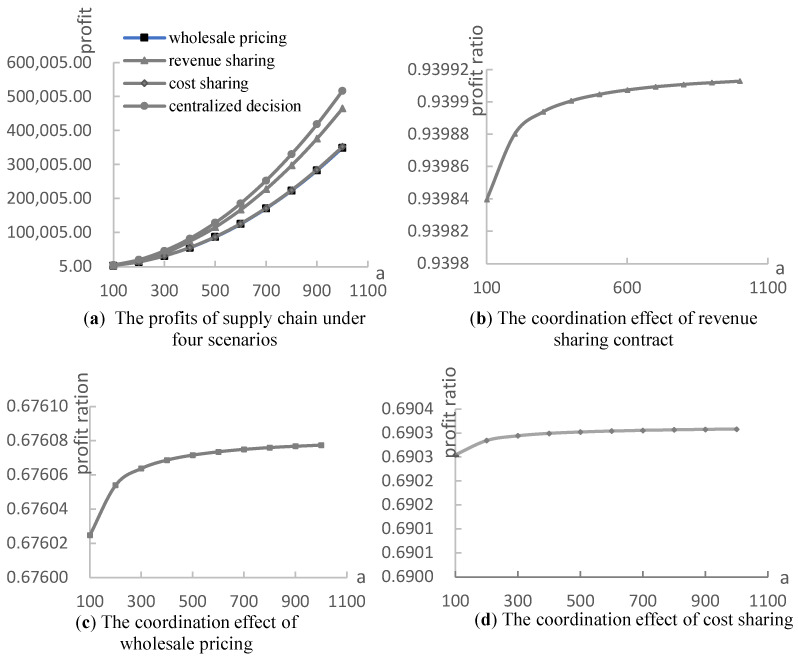
The effect of the consumers with environmental awareness on the profit and coordination of the supply chain.

Finally, the effect of the consumers with environmental awareness (green consumers) was employed using numerical experiments with the assumptions a∈[100,1000],η=5,α=0.3,β=0.5,s=0.5, the optimal λ and k, and are shown in Figure 8 and Figure 9. Because it is assumed that consumers in the market have environmental awareness and the sensitivity coefficient of consumers to product greenness is 1, this section analyzes the impact of the initial market potential on the decisions and profits of the supply chain. From Figure 8a, we can observe that the greenness of two complementary products increases with the market capacity of consumers with environmental awareness. Furthermore, it can be observed that the effect of green subsidies on the greenness of a supply chain with a=300 is higher than that with a=100, as is presented in Figure 8b. Therefore, more consumers with environmental awareness improve the efficiency of government subsidies to the green supply chain. Additionally, from Figure 9a, we can determine that the profits of the supply chain under four scenarios increase with the capacity of the green consumers. Figure 9b–d shows that the ratio of the supply chain profit under three contracts to centralized decision scenarios increases with the capacity of the green consumers. The higher ratio means a better coordination of the supply chain. In other words, a large number of green consumers also improves the coordination effect of the supply chain contracts, such as revenue sharing and cost sharing. To summarize, improving consumers’ awareness of environmental protection and increasing the scale of green consumers in the market are conducive to the development of the green supply chain and environmental protection.

## 6. Conclusions

In the context of green economy development, increasingly more enterprises are encouraged to implement green innovation and pay attention to green supply chain management, such as Huawei, HP, Apple Inc and Procter & Gamble. On the other hand, with the increasingly fierce market competition, complementary product strategy and complementary product supply chain management have attracted much attention. However, due to the spillover effect of green innovation, complementary products inevitably play an important role in the development of the green economy. However, in theory, very little research has discussed the decisions and contract of the green complementary product supply chain. In order to appreciate the use of complementary product strategies in a low-carbon economy, this paper constructs game models for the complementary product supply chain considering government green subsidies presented to retailers for product greenness and consumers’ environmental awareness. To solve the models, the members’ optimal product greenness and pricing decisions under the wholesale price contract and subsidy-, revenue- and cost-sharing contracts are analyzed. Then, the impact of the contracts on the greenness and performance of the complementary product supply chain is discussed.

According to the results, our main findings are as follows. Firstly, in the centralized and decentralized complementary product supply chain (under all contracts), government green subsidies and green consumers with environmental awareness encourage manufacturers to increase the greenness of both complementary products and play a very positive role in environmental protection. On the market side, consumers’ environmental awareness can also encourage manufacturers to improve the green degree of both complementary products. Moreover, the incentive degree from the government and consumers increases with the spillover utility between the complementary products. Therefore, in the process of achieving a carbon peak and carbon neutralization, the government and the leaders of the supply chain could use the complementary product strategy to improve the efficiency of green innovation, such as actively guiding consumers’ green consumption and creating the green spillover effect of complementary products. Secondly, in comparison to the wholesale price contract, both the revenue- and cost-sharing contracts can not only incentivize manufacturers to increase the supply chain greenness and subsidized product greenness, but also achieve the Pareto improvement for the supply chain members, if the contract parameters are appropriate, which is conducive to supply chain optimization, while the revenue-sharing contract is more effective. In the above sharing contract, retailers could gain a greater profit through eliminating the double marginal utility in the decentralized supply chain by sharing revenues and costs with upstream manufacturers, leading to the improvement of other members and supply chains at the same time. Therefore, the concept of sharing is often a win–win situation. Thirdly, unexpectedly, the retailer’s subsidy-sharing contract cannot incentivize manufacturers to increase product greenness, but only changes the manufacturer’s wholesale price of the subsidized products and affects the profit distribution between the manufacturer and the retailer. Another issue to note is that the manufacturers reduce the greenness of product 2 under revenue-sharing contracts, although its coordination effect is the best overall.

In summary, we expand on the research on the complementary product supply chain focusing on green decisions and contracts. Indeed, we provide marketing models to consider how sharing contracts and the complementarity of products affect the green innovation of channel members and the performance of the supply chain. We also put forward valuable insights into the importance of government green subsidies for and consumers’ awareness of the supply chain management, which is beneficial to the green development of the supply chain and the carbon neutrality achievement of countries.

Certainly, our article still has some shortcomings and there are several research directions that might be explored in future research. First, this paper only considers one form of government subsidy to retailers for greenness, while, in practice, government subsidies take various forms, such as one-time input subsidies for manufacturers and purchase subsidies for consumers. Therefore, it would be valuable to examine the influence of different government subsidies on the green innovation decision of the complementary product supply chain. Second, in addition to subsidies, the government also uses carbon emission restrictions, green certification, and green labels to encourage supply chain members to practice green manufacturing. Therefore, it is necessary and meaningful to study further the optimization of complementary product supply chains under the government’s green incentives and restrictions.

## Data Availability

Not applicable.

## Appendix A

$$A_{1}=8\left(1+\lambda \right){\left(1-\alpha^{2}\right)}^{2}\lambda ,$$

$$\begin{array}{c} B_{1}=2{\left(1+\lambda \right)}^{2}{\left(1-\alpha^{2}\right)}^{2}\alpha \lambda +2\left(1-\alpha^{2}\right)\left(1-\lambda^{2}\right)\alpha \lambda +\left(1+\lambda \right)\left(1-\alpha^{2}\right)\alpha \lambda [4\lambda -\left(1+\lambda {)}^{2}\alpha^{2}\right],\\ C_{1}=2(1+\lambda )(1-\alpha^{2})\lambda \{[2\lambda -(1+\lambda )\alpha^{2}\lambda ](1-s)-\alpha (1-\lambda )(\beta -\alpha \lambda s)\}+\\ 2(1-\alpha^{2})\lambda [2\lambda (1+s)-{\left(1+\lambda \right)}^{2}\alpha^{2}s-\alpha (1+\lambda )(\beta -\alpha \lambda s)](1-\lambda )-2(1+\lambda )(1-\alpha^{2})\lambda [4\lambda -{\left(1+\lambda \right)}^{2}\alpha^{2}]\\ D_{1}=2(1+\lambda )(1-\alpha^{2})\lambda [2(\alpha +\beta )\lambda -(1+\lambda )(1+\alpha \beta \lambda )\alpha ]+2(1-\alpha^{2})\lambda [2\lambda \beta -(1+\lambda )\alpha ](1-\lambda )-2(1+\lambda )(1-\alpha^{2})\lambda [4\lambda -{\left(1+\lambda \right)}^{2}\alpha^{2}] \end{array}$$

$$E_{1}=2(1+\lambda )(1-\alpha^{2})\lambda [4\lambda -(1+\lambda {)}^{2}\alpha^{2}]a-2(1+\lambda )(1-\alpha^{2})\lambda (1+\alpha )[2\lambda -(1+\lambda )\alpha ]a-2(1-\alpha^{2})\lambda [2\lambda -(1+\lambda )\alpha ](1-\lambda )a$$

$$A_{2}=2{\left(1+\lambda \right)}^{2}{\left(1-\alpha^{2}\right)}^{2}\alpha \lambda +2(1-\alpha^{2})(1-\lambda^{2})\alpha \lambda +(1+\lambda )(1-\alpha^{2})\alpha \lambda [4\lambda -{\left(1+\lambda \right)}^{2}\alpha^{2}]$$

$$B_{2}=2{\left(1-\lambda \right)}^{2}(1+\lambda )(1-\alpha^{2})\alpha +4[4\lambda -{\left(1+\lambda \right)}^{2}\alpha^{2}](1-\alpha^{2})\lambda$$

$$\begin{array}{l} C_{2}={\left(1-\lambda \right)}^{2}\alpha \{[2\lambda -(1+\lambda )\alpha^{2}\lambda ](1-s)-\alpha (1-\lambda )(\beta -\alpha \lambda s)\}+(1+\lambda )(1-\alpha^{2})\alpha [2\lambda (1+s)-{\left(1+\lambda \right)}^{2}\alpha^{2}s-\alpha (1+\lambda )(\beta -\alpha \lambda s)](1-\lambda )+\\ {}[4\lambda -{\left(1+\lambda \right)}^{2}\alpha^{2}]\{[2\lambda -(1+\lambda )\alpha^{2}\lambda ](\beta -\alpha \lambda s)-\alpha \lambda (1-s)\}-{\left(1-\lambda \right)}^{2}\alpha [4\lambda -{\left(1+\lambda \right)}^{2}\alpha^{2}]-{\left[4\lambda -{\left(1+\lambda \right)}^{2}\alpha^{2}\right]}^{2} \end{array}$$

$$\begin{array}{l} D_{2}={\left(1-\lambda \right)}^{2}\alpha [2(\alpha +\beta )\lambda -(1+\lambda )\alpha (1+\alpha \beta \lambda )]+(1+\lambda )\alpha (1-\alpha^{2})[2\beta \lambda -(1+\lambda )\alpha ](1-\lambda )+\\ {}[4\lambda -{\left(1+\lambda \right)}^{2}\alpha^{2}][2\lambda +(1-\lambda )\alpha \beta \lambda -(1+\lambda )\alpha^{2}]-{\left(1-\lambda \right)}^{2}\alpha [4\lambda -{\left(1+\lambda \right)}^{2}\alpha^{2}]\beta -{\left[4\lambda -{\left(1+\lambda \right)}^{2}\alpha^{2}\right]}^{2}\beta \end{array}$$

$$\begin{array}{l} E_{2}={\left(1-\lambda \right)}^{2}\alpha [4\lambda -{\left(1+\lambda \right)}^{2}\alpha^{2}]a-{\left(1-\lambda \right)}^{2}\alpha \{(1+\alpha )[2\lambda -(1+\lambda )\alpha ]\}a-(1+\lambda )(1-\alpha^{2})\alpha [2\lambda -(1+\lambda )\alpha ](1-\lambda )a+\\ {\left[4\lambda -{\left(1+\lambda \right)}^{2}\alpha^{2}\right]}^{2}a-[4\lambda -{\left(1+\lambda \right)}^{2}\alpha^{2}](1+\alpha )[2\lambda -(1+\lambda )\alpha ]a \end{array}$$

$$A_{3}=[2\lambda (1+s)-{\left(1+\lambda \right)}^{2}\alpha^{2}s-\alpha (1+\lambda )(\beta -\alpha \lambda s)](1-\lambda )2(1-\alpha^{2})\lambda -2(1+\lambda )\lambda (1-\alpha^{2})\{4\lambda -{\left(1+\lambda \right)}^{2}\alpha^{2}-[2\lambda -(1+\lambda )\alpha^{2}\lambda ](1-s)+\alpha (1-\lambda )(\beta -\alpha \lambda s)\}$$

$$\begin{array}{l} B_{3}=[2\lambda (1+s)-{\left(1+\lambda \right)}^{2}\alpha^{2}s-\alpha (1+\lambda )(\beta -\alpha \lambda s)](1-\lambda )(1-\alpha^{2})(1+\lambda )\alpha -{\left(1-\lambda \right)}^{2}\alpha \{4\lambda -{\left(1+\lambda \right)}^{2}\alpha^{2}-[2\lambda -(1+\lambda )\alpha^{2}\lambda ](1-s)+\alpha (1-\lambda )(\beta -\alpha \lambda s)\}-\\ \left[4\lambda -{\left(1+\lambda \right)}^{2}\alpha^{2}]\{[4\lambda -{\left(1+\lambda \right)}^{2}\alpha^{2}]\beta -[2\lambda -(1+\lambda )\alpha^{2}](\beta -\alpha \lambda s)+\alpha \lambda (1-\lambda )\right\} \end{array}$$

$$C_{3}=[2\lambda (1+s)-{\left(1+\lambda \right)}^{2}\alpha^{2}s-\alpha (1+\lambda )(\beta -\alpha \lambda s)](1-\lambda )\{2[[2\lambda -(1+\lambda )\alpha^{2}\lambda ](1-s)+\alpha (1-\lambda )(\beta -\alpha \lambda s)]-2[4\lambda -{\left(1+\lambda \right)}^{2}\alpha^{2}]\}+\eta {\left[4\lambda -{\left(1+\lambda \right)}^{2}\alpha^{2}\right]}^{2}$$

$$\begin{array}{l} D_{3}=[2\lambda (1+s)-{\left(1+\lambda \right)}^{2}\alpha^{2}s-\alpha (1+\lambda )(\beta -\alpha \lambda s)](1-\lambda )\{2(\alpha +\beta )\lambda -(1+\lambda )(1+\alpha \beta \lambda )\alpha -\beta [4\lambda -{\left(1+\lambda \right)}^{2}\alpha^{2}]\}-\\ \left\{4\lambda -{\left(1+\lambda \right)}^{2}\alpha^{2}-[2\lambda -(1+\lambda )\alpha^{2}\lambda ](1-s)+\alpha (1-\lambda )(\beta -\alpha \lambda s)\}[2\beta \lambda -(1+\lambda )\alpha ](1-\lambda \right) \end{array}$$

$$\begin{array}{l} E_{3}=[2\lambda (1+s)-{\left(1+\lambda \right)}^{2}\alpha^{2}s-\alpha (1+\lambda )(\beta -\alpha \lambda s)](1-\lambda )\{[4\lambda -{\left(1+\lambda \right)}^{2}\alpha^{2}]a-(1+\alpha )[2\lambda -(1+\lambda )\alpha ]a\}+\\ \{4\lambda -{\left(1+\lambda \right)}^{2}\alpha^{2}-[2\lambda -(1+\lambda )\alpha^{2}\lambda ](1-s)+\alpha (1-\lambda )(\beta -\alpha \lambda s)\}[2\lambda -(1+\lambda )\alpha ](1-\lambda )a \end{array}$$

$$A_{4}=2[2\beta \lambda -(1+\lambda )\alpha ](1-\lambda )(1-\alpha^{2})\lambda -\{\beta [4\lambda -{\left(1+\lambda \right)}^{2}\alpha^{2}]-2(\alpha +\beta )\lambda +(1+\lambda )\alpha (1+\alpha \beta \lambda )\}2(1+\lambda )(1-\alpha^{2})\lambda$$

$$\begin{array}{l} B_{4}=[2\beta \lambda -(1+\lambda )\alpha ](1-\lambda )(1+\lambda )(1-\alpha^{2})\alpha -\{\beta [4\lambda -{\left(1+\lambda \right)}^{2}\alpha^{2}]-2(\alpha +\beta )\lambda +(1+\lambda )\alpha (1+\alpha \beta \lambda )\}{\left(1-\lambda \right)}^{2}\alpha -\\ \left[4\lambda -{\left(1+\lambda \right)}^{2}\alpha^{2}][4\lambda -{\left(1+\lambda \right)}^{2}\alpha^{2}-2\lambda -(1-\lambda )\alpha \beta \lambda +(1+\lambda )\alpha^{2}\right] \end{array}$$

$$\begin{array}{l} C_{4}=[2\beta \lambda -(1+\lambda )\alpha ](1-\lambda )\{[2\lambda -(1+\lambda )\alpha^{2}\lambda ](1-s)-\alpha (1-\lambda )(\beta -\alpha \lambda s)-[4\lambda -{\left(1+\lambda \right)}^{2}\alpha^{2}]\}-\\ \left\{\beta [4\lambda -{\left(1+\lambda \right)}^{2}\alpha^{2}]-2(\alpha +\beta )\lambda +(1+\lambda )\alpha (1+\alpha \beta \lambda )\}[2\lambda (1+s)-{\left(1+\lambda \right)}^{2}\alpha^{2}s-\alpha (1+\lambda )(\beta -\alpha \lambda s)](1-\lambda \right) \end{array}$$

$$D_{4}=[2\beta \lambda -(1+\lambda )\alpha ](1-\lambda )\{2[2(\alpha +\beta )\lambda -(1+\lambda )(1+\alpha \beta \lambda )\alpha ]-2\beta [4\lambda -{\left(1+\lambda \right)}^{2}\alpha^{2}]\}+\eta {\left[4\lambda -{\left(1+\lambda \right)}^{2}\alpha^{2}\right]}^{2}$$

$$\begin{array}{l} E_{4}=[2\beta \lambda -(1+\lambda )\alpha ](1-\lambda )\{[4\lambda -{\left(1+\lambda \right)}^{2}\alpha^{2}]a-(1+\alpha )[2\lambda -(1+\lambda )\alpha ]a\}+\\ \left\{\beta [4\lambda -{\left(1+\lambda \right)}^{2}\alpha^{2}]-2(\alpha +\beta )\lambda +(1+\lambda )\alpha (1+\alpha \beta \lambda )\}\{[2\lambda -(1+\lambda )\alpha ](1-\lambda )a\right\} \end{array}$$

## Appendix B

**Table A1:** The data of Figure 2a.

	*α* = 0.2		*α* = 0.25		*α* = 0.3		*α* = 0.35	
***s***	*e*1	*e*2	*e*1	*e*2	*e*1	*e*2	*e*1	*e*2
**0.1**	16.50739	16.04563	15.80854	15.44178	15.18297	14.90531	14.62391	14.43188
**0.2**	17.34231	16.9449	16.59817	16.29927	15.9317	15.72551	15.33546	15.21875
**0.3**	18.20924	17.8979	17.41632	17.20694	16.70574	16.59265	16.06929	16.04956
**0.4**	19.10569	18.90446	18.26034	18.16438	17.50227	17.50607	16.82241	16.92338
**0.5**	20.0282	19.9635	19.12664	19.17026	18.31757	18.46422	17.591	17.83843
**0.6**	20.97221	21.07281	20.01058	20.22217	19.14696	19.46446	18.37029	18.79188
**0.7**	21.93197	22.22889	20.90643	21.31643	19.98469	20.50299	19.15452	19.77974
**0.8**	22.90042	23.42674	21.80721	22.44797	20.82388	21.57462	19.93693	20.79675
**0.9**	23.86915	24.65968	22.70476	23.61014	21.65659	22.67273	20.70976	21.83626
**1**	24.82838	25.91931	23.58967	24.79466	22.47377	23.78918	21.46431	22.89024

**Table A2:** The data of Figure 2b and Figure 3.

	*β* = 0.5		*β* = 0.55		*β* = 0.6		*β* = 0.65	
***s***	*e*1	*e*2	*e*1	*e*2	*e*1	*e*2	*e*1	*e*2
**0.1**	15.18297	14.90531	15.97515	15.59894	16.80254	16.31892	17.66809	17.06746
**0.2**	15.9317	15.72551	16.77289	16.47224	17.653	17.24868	18.57543	18.05745
**0.3**	16.70574	16.59265	17.59826	17.39709	18.53378	18.23502	19.51616	19.10954
**0.4**	17.50227	17.50607	18.44835	18.37299	19.44186	19.27765	20.48722	20.22366
**0.5**	18.31757	18.46422	19.31927	19.3985	20.37323	20.37525	21.48446	21.39872
**0.6**	19.14696	19.46446	20.20612	20.47104	21.32276	21.52534	22.50253	22.63233
**0.7**	19.98469	20.50299	21.10284	21.58675	22.28407	22.72404	23.53479	23.92064
**0.8**	20.82388	21.57462	22.00218	22.74031	23.24953	23.96589	24.57319	25.25809
**0.9**	21.65659	22.67273	22.8957	23.92482	24.2102	25.24375	25.60825	26.63729
**1**	22.47377	23.78918	23.7738	25.13173	25.15587	26.54863	26.62914	28.04884

**Table A3:** The data of Figure 4 and Figure 5.

Wholesale Price							
*s*	*e*1	*e*2	*e1 + e*2	Profit of Manufacture	Profit of Retailer	Profit of Supply Chain	△
**0.1**	6.938439	6.356651	13.29509	2123.506	1172.439	3295.945	42.80052
**0.2**	7.583779	6.401738	13.98552	2143.751	1194.995	3338.746	47.66912
**0.3**	8.254836	6.451366	14.7062	2166.141	1220.274	3386.415	53.00053
**0.4**	8.954975	6.505894	15.46087	2190.845	1248.57	3439.415	58.87407
**0.5**	9.687962	6.565738	16.2537	2218.055	1280.234	3498.29	65.38423
**0.6**	10.45804	6.631381	17.08942	2247.994	1315.679	3563.674	72.6444
**0.7**	11.27003	6.703378	17.9734	2280.921	1355.397	3636.318	80.7918
**0.8**	12.12941	6.782376	18.91179	2317.137	1399.973	3717.11	89.99387
**0.9**	13.04252	6.869129	19.91164	2356.992	1450.111	3807.104	100.4567
**1**	14.01664	6.96452	20.98117	2400.898	1506.663	3907.561	
**Cost Sharing**							
***λ***	*S*	*e*1	*e*2	*e*1 + *e*2	Profit of Manufacture	Profit of Retailer	Profit of Supply Chain
**0.6**	0.1	8.197436	5.779014	13.97645	2745.515	1393.949	4139.465
**0.6**	0.2	8.972817	5.858294	14.83111	2782.343	1418.571	4200.913
**0.6**	0.3	9.783773	5.944864	15.72864	2823.049	1446.661	4269.71
**0.5**	0.4	11.47491	5.934722	17.40963	3074.515	1481.68	4556.195
**0.5**	0.5	12.45963	6.04583	18.50546	3132.601	1521.988	4654.589
**0.5**	0.6	13.50127	6.167774	19.66904	3196.998	1567.799	4764.798
**0.5**	0.7	14.60743	6.301728	20.90916	3268.484	1619.916	4888.4
**0.5**	0.8	15.78695	6.449066	22.23601	3347.985	1679.312	5027.298
**0.5**	0.9	17.05009	6.611407	23.6615	3436.613	1747.176	5183.789
**0.5**	1	18.40895	6.790665	25.19961	3535.708	1824.967	5360.676
**Revenue Sharing**							
***s***	*k*	*e*1	*e*2	*e*1 + *e*2	Profit of Manufacture	Profit of Retailer	Profit of Supply Chain
**0.1**	0.05	7.330056	6.371873	13.70193	2129.863	1172.771	3302.634
**0.2**	0.05	8.01755	6.419743	14.43729	2151.351	1195.491	3346.842
**0.3**	0.07	8.94155	6.481681	15.42323	2179.058	1220.996	3400.054
**0.4**	0.07	9.712287	6.541323	16.25361	2206.065	1249.668	3455.734
**0.5**	0.07	10.52212	6.606963	17.12908	2235.894	1281.843	3517.737
**0.6**	0.09	11.66903	6.694423	18.36346	2275.452	1317.997	3593.449
**0.7**	0.09	12.60382	6.77633	19.38015	2312.882	1358.795	3671.677
**0.8**	0.09	13.59927	6.866648	20.46592	2354.251	1404.822	3759.073
**0.9**	0.11	15.08072	6.991362	22.07208	2411.082	1457.111	3868.193
**1**	0.11	16.2705	7.105631	23.37613	2463.614	1516.63	3980.244
**Centralized Decision**							
***s***		*e*1	*e*2	*e*1 + *e*2	Profit of Supply Chain	△	
**0.1**		15.18297	14.90531	30.08828	4739.827	96.51116	
**0.2**		15.9317	15.72551	31.65721	4836.338	104.2811	
**0.3**		16.70574	16.59265	33.29839	4940.619	112.6213	
**0.4**		17.50227	17.50607	35.00834	5053.24	121.5547	
**0.5**		18.31757	18.46422	36.78179	5174.795	131.0931	
**0.6**		19.14696	19.46446	38.61142	5305.888	141.2308	
**0.7**		19.98469	20.50299	40.48767	5447.119	151.9368	
**0.8**		20.82388	21.57462	42.3985	5599.056	163.1453	
**0.9**		21.65659	22.67273	44.32932	5762.201	174.745	
**1**		22.47377	23.78918	46.26295	5936.946		

**Table A4:** The data of Figure 6.

*λ*	*e*1	*e*2	Profit of Manufacture	Profit of Retailer	Profit of Supply Chain
**0.2**	21.19551	4.512006	5511.877	122.9486	5634.826
**0.3**	19.26725	6.118647	4869.408	1308.865	6178.274
**0.4**	17.32611	6.370909	4332.159	1612.607	5944.766
**0.5**	15.78695	6.449066	3929.622	1679.312	5608.934
**0.6**	14.56599	6.515504	3609.741	1657.033	5266.774
**0.7**	13.58391	6.604719	3340.806	1595.819	4936.626
**0.8**	12.78583	6.723185	3103.803	1516.746	4620.549
**0.9**	12.13388	6.869946	2886.769	1429.965	4316.734
**0.95**	11.854	6.953052	2783.177	1385.456	4168.633
**1**	11.60122	7.042105	2681.861	1340.791	4022.653

**Table A5:** The data of Figure 7.

*k*	*e*1	*e*2	Profit of Manufacture	Profit of Retailer	Profit of Supply Chain
**0.01**	12.27685	6.790829	2320.86	1400.823	3721.683
**0.03**	12.58274	6.808367	2328.584	1402.329	3730.913
**0.05**	12.90427	6.826801	2336.702	1403.535	3740.238
**0.07**	13.24266	6.846202	2345.247	1404.387	3749.634
**0.09**	13.59927	6.866648	2354.251	1404.822	3759.073
**0.11**	13.97563	6.888226	2363.754	1404.766	3768.52
**0.13**	14.3734	6.911032	2373.798	1404.13	3777.929
**0.15**	14.79449	6.935174	2384.43	1402.814	3787.244
**0.17**	15.24099	6.960773	2395.705	1400.694	3796.398
**0.19**	15.71528	6.987966	2407.68	1397.626	3805.306

**Table A6:** The data of Figure 8 and Figure 9.

Wholesale Price					
***a***	*e*1	*e*2	*e*1 + *e*2	Profit of Supply Chain	Profit Ratio
**100**	9.687962	6.565738	16.2537	3498.29	0.676025
**200**	19.37592	13.13148	32.5074	13,993.16	0.676054
**300**	29.06389	19.69722	48.7611	31,484.61	0.676064
**400**	38.75185	26.26295	65.0148	55,972.63	0.676069
**500**	48.43981	32.82869	81.2685	87,457.24	0.676072
**600**	58.12777	39.39443	97.5222	125,938.4	0.676074
**700**	67.81574	45.96017	113.7759	171,416.2	0.676075
**800**	77.5037	52.52591	130.0296	223,890.5	0.676076
**900**	87.19166	59.09165	146.2833	283,361.5	0.676077
**1000**	96.87962	65.65738	162.537	349,829	0.676077
**Revenue Sharing**					
***a***	*e*1	*e*2	*e*1 + *e*2	Profit of Supply Chain	Profit Ratio
**100**	15.20804	5.626822	20.83486	4863.478	0.93984
**200**	30.41609	11.25364	41.66973	19,453.91	0.93988
**300**	45.62413	16.88047	62.50459	43,771.3	0.939894
**400**	60.83217	22.50729	83.33946	77,815.64	0.939901
**500**	76.04022	28.13411	104.1743	121,586.9	0.939905
**600**	91.24826	33.76093	125.0092	175,085.2	0.939907
**700**	106.4563	39.38775	145.8441	238,310.4	0.939909
**800**	121.6643	45.01457	166.6789	311,262.6	0.939911
**900**	136.8724	50.6414	187.5138	393,941.7	0.939912
**1000**	152.0804	56.26822	208.3486	486,347.8	0.939913
**Cost Sharing**					
***a***	*e*1	*e*2	*e*1 + *e*2	Profit of Supply Chain	Profit Ratio
**100**	14.6733	6.812115	21.48542	3571.926	0.690255
**200**	29.3466	13.62423	42.97083	14,287.7	0.690284
**300**	44.0199	20.43635	64.45625	32,147.33	0.690294
**400**	58.6932	27.24846	85.94166	57,150.81	0.690299
**500**	73.3665	34.06058	107.4271	89,298.14	0.690302
**600**	88.0398	40.87269	128.9125	128,589.3	0.690304
**700**	102.7131	47.68481	150.3979	175,024.4	0.690306
**800**	117.3864	54.49692	171.8833	228,603.2	0.690307
**900**	132.0597	61.30904	193.3687	289,326	0.690308
**1000**	146.733	68.12115	214.8542	357,192.6	0.690308
**Centralized Decision**					
***a***	*e*1	*e*2	*e*1 + *e*2	Profit of Supply Chain	
**100**	18.31757	18.46422	36.78179	5174.795	
**200**	36.61361	36.92843	73.54204	20,698.28	
**300**	54.90965	55.39265	110.3023	46,570.47	
**400**	73.20569	73.85686	147.0626	82,791.35	
**500**	91.50173	92.32108	183.8228	129,360.9	
**600**	109.7978	110.7853	220.5831	186,279.2	
**700**	128.0938	129.2495	257.3433	253,546.1	
**800**	146.3899	147.7137	294.1036	331,161.8	
**900**	164.6859	166.1779	330.8638	419,126.1	
**1000**	182.9819	184.6422	367.6241	517,439.2	
